# An integrated study discloses chopping tools use from Late Acheulean Revadim (Israel)

**DOI:** 10.1371/journal.pone.0245595

**Published:** 2021-01-19

**Authors:** Flavia Venditti, Aviad Agam, Jacopo Tirillò, Stella Nunziante-Cesaro, Ran Barkai

**Affiliations:** 1 Department of Early Prehistory and Quaternary Ecology, University of Tübingen, Tübingen, Germany; 2 Department of Classics, LTFAPA Laboratory, “Sapienza”, University of Rome, Rome, Italy; 3 Institute of Archaeology, Tel Aviv University, Tel Aviv, Israel; 4 Department of Chemical Engineering Materials Environment, University of Rome “Sapienza”, University of Rome, Italy; 5 Scientific Methodologies Applied to Cultural Heritage (SMATCH), Rome, Italy; Universita degli Studi di Ferrara, ITALY

## Abstract

Chopping tools/choppers provide one of the earliest and most persistent examples of stone tools produced and used by early humans. These artifacts appeared for the first time ~2.5 million years ago in Africa and are characteristic of the Oldowan and Acheulean cultural complexes throughout the Old World. Chopping tools were manufactured and used by early humans for more than two million years regardless of differences in geography, climate, resource availability, or major transformations in human cultural and biological evolution. Despite their widespread distribution through time and space in Africa and Eurasia, little attention has been paid to the function of these items, while scholars still debate whether they are tools or cores. In this paper, we wish to draw attention to these prominent and ubiquitous early lithic artifacts through the investigation of 53 chopping tools retrieved from a specific context at Late Acheulean Revadim (Israel). We combined typo-technological and functional studies with a residue analysis aimed at shedding light on their functional role within the tool-kits of the inhabitants of the site. Here we show that most of the chopping tools were used to chop hard and medium materials, such as bone, most probably for marrow extraction. A few of the tools were also used for cutting and scraping activities, while some also served as cores for further flake detachment. The chopping tools exhibit extraordinarily well-preserved bone residues suggesting they were used mainly for bone-breaking and marrow acquisition. We discuss the data and explore the tool versus core debate also in light of a sample of 50 flake cores made on pebbles/cobbles retrieved from the same archeological layer. The results add further pieces to the puzzle of activities carried out at Revadim and add to our knowledge of the production and use of these enigmatic tools and their role in human evolutionary history.

## Introduction

Choppers and chopping tools are one of the most enduring and persistent categories of stone artifacts produced and used by early humans. These items appear in the archaeological record as early as 2.6 Ma in Africa and persist until 500–300 ka in Asia and Europe [1–11], predating the canonical Lower Paleolithic handaxes [see 12 for an overview].

Given their widespread distribution through time and space, choppers and chopping tools have been discussed in several studies concerning Lower Paleolithic assemblages [e.g., 13–18]. Despite of that, a lack of sufficient criteria has hindered their secure classification either as cores or formal tools and many scholars preferred to classify them as both core and tool [14].

According to Leakey [19], a chopper is a core-tool with a flaked edge on one or two intersected faces. It is unifacial if the edge was formed by flaking only on one face of the item, and bifacial if both faces were flaked. Bifacially flaked core-forms (namely a worked nodule/pebble as opposed to a worked flake/blade) were classified by Movius [20] as chopping tools. Keeley [21] and Singer [18] defined chopping tools as “chopper-cores” while Semenov [22] coined the term “core-like chopping tool”, a term used at that time in the Western and Soviet archeological literature.

The characterization of a *core form* as either *core* or *core tool* is very subtle and is still debated among scholars [for an overview on this topic, see 23 and contributions therein].

M. Leakey was the first to typologically characterize core-tool forms (pebbles and angular rock fragments from which flakes were removed) such as choppers and heavy-duty scrapers as “heavy-duty tools” [19, 24, 25]. She, however, classified Oldowan core-tools on the basis of morphology alone, with no data regarding their function, notwithstanding her assumption that most of the core-forms were deliberately produced to be used as designated tools. Advocating a different approach, Isaac [26] and Toth [13, 27] argued that “core-forms” in Oldowan assemblages were by-products (namely cores) resulting from the production of sharp usable cutting flakes rather than being the target forms. Although Toth does not deny the possibility that some “core-forms” were used as tools, he also remarks that heavy-duty tasks are easily performed with unshaped cobbles [13].

Keeley and Toth argued that core-tools can be the result of flaking stone cobbles or chunks mainly to produce sharp stone flakes. This assumption was supported, to some extent, by a study of 54 fine-grained flakes and flake fragments from Koobi Fora, Kenya (1.9–1.4 million years ago), which showed the presence of diagnostic polishes on 9 flakes [28]. Moreover, experiments performed by Toth [13], demonstrated that all varieties of core-forms, long assumed to be deliberately shaped "tools", can result from flaking stone cobbles or chunks to produce sharp flakes.

The idea that items classified as choppers and chopping tools are in fact cores used for flake production is not new and has been advocated for several decades [13, 23, 26–27, 29]. It is further supported by the little evidence of use-wear related to chopping activities using chopping tools [14].

The first detailed microwear analysis of core-chopping tool was Keeley’s study of flaked stone artifacts from Clacton and Hoxne [21]. He analyzed 22 chopper-cores from Clacton and found reliable wear on only 2 artefacts which were interpreted as having been used to chop wood [21:116]. Two other chopper-cores from Hoxne displayed traces of bone and were interpreted as having been used to open up bone for marrow extraction [21:140, 146].

Chopping tools have been frequently associated with bone breaking and heavy duty-wood working [13, 21, 30, 31], but no extensive functional microscopic examination for this class of artefacts has been conducted in recent years.

With the aim of filling this gap, this work discusses the technology and function of these ubiquitous early lithic artifacts presenting an integrated techno-functional and residue analysis on 53 chopping tools and 50 pebble cores. The analyzed material was recovered from Area C Layer 3 at the Late Acheulean site of Revadim (Israel). We explore the role of chopping tools within the lithic assemblage and interpret them in light of results from a sample of flake cores produced on pebbles (with one and two striking platforms). Our integrated approach combines flint type characterization, typo-technological classification, experimental observations, use-wear analysis and morphological observations combined with elemental and chemical analysis of residues. The results show that a great percentage of chopping tools were used for pounding activities on hard and medium materials with direct correspondence between use-wear and residues related to bone processing activities. It is therefore suggested that in the case of Revadim, chopping tools were mostly used as designated tools aimed at marrow extraction. A few of the tools also served for activities of scraping and cutting, thus highlighting the potential versatility of these artefacts.

## Archaeological context

**Fig 1:**
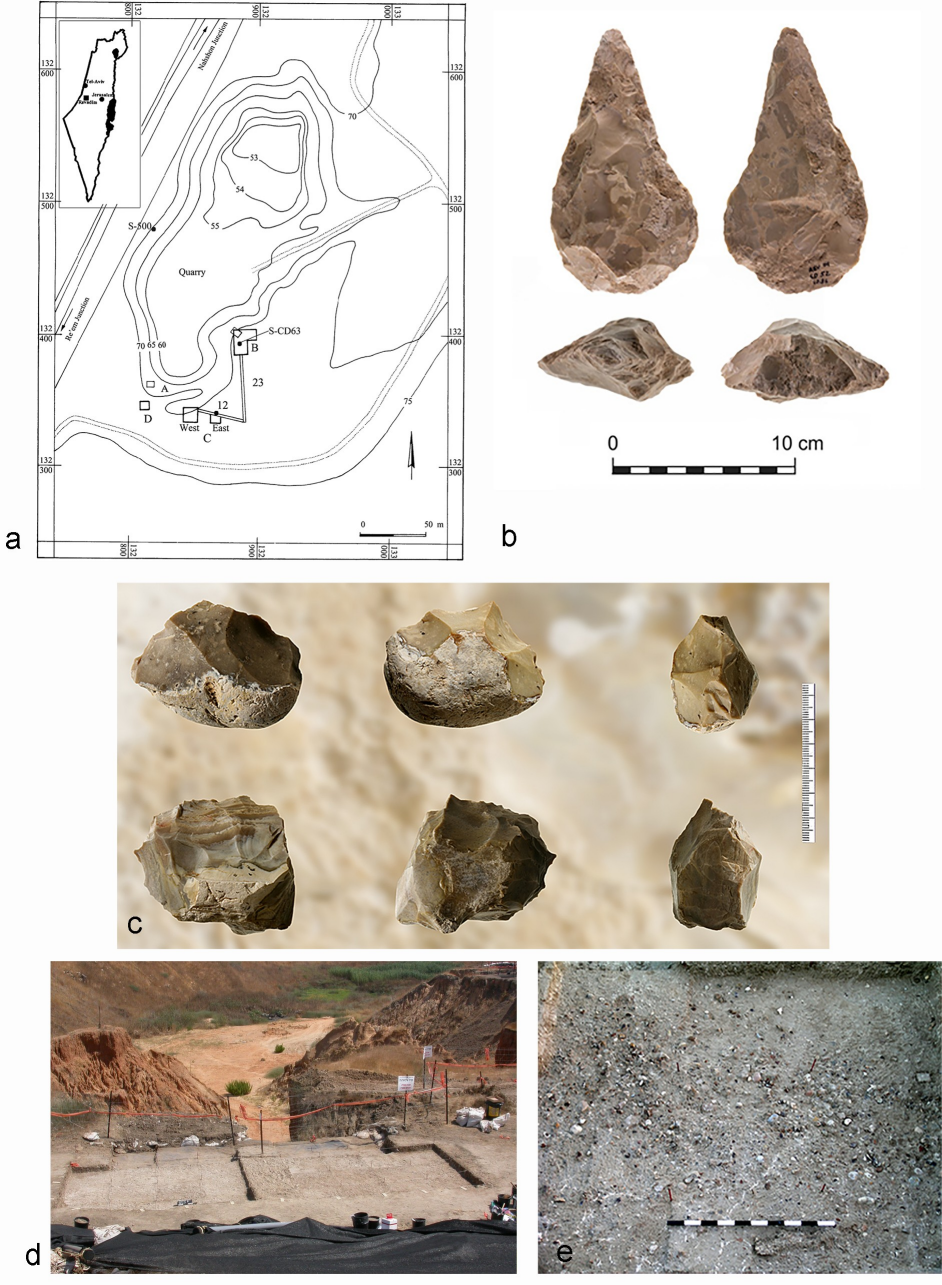
Geographical and archaeological setting of Revadim site. a) Site location and excavation areas of late Acheulean Revadim; b) A handaxe from Area C Layer 3; c) Chopping tools from Area C; d) Area C, view to the northeast; e) Close-up of Layer C3.

Revadim is an open-air site located on the southern Coastal Plain of Israel, ~40 km southeast of Tel-Aviv, 71–73 m above sea level [32] (Fig 1A). Several excavation seasons were carried out between 1996 and 2004 on behalf of the Israel Antiquities Authority and the Hebrew University of Jerusalem, under the direction of O. Marder and I. Milevski [32, 33].

The site was preliminarily dated using paleomagnetic analyses of the geological sequence. The results demonstrated normal polarity, showing that the entire sequence is younger than 780,000 years [33]. Uranium series dating of carbonates covering flint items was also conducted, providing a minimum age of between 300,000 and 500,000 years [34]. The lithic assemblages are dominated by flakes and flake-tools, along with bifaces, chopping tools and flake-cores [35–37] (Fig 1B and 1C).

The faunal assemblage of Revadim has yielded thousands of animal bones. Faunal analysis was performed mainly on the Area B assemblage and was focused on the elephant bones, while Area C was investigated to a lesser extent [36, 38, 39]. The faunal assemblage consists mainly of bone splinters and the represented animal taxa include *Palaeoloxodon antiquus*, *Bos primigenius*, *Dama cf*. *mesopotamica*, *Cervus elaphus*, *Capreolus cf*. *capreolus* in addition to other mammals such as wild boar (*Sus scrofa*), equids and micro-vertebrates. The major accumulation of elephant bones was retrieved from Area B, where at least three individuals were identified, while an elephant skull fragment, a part of a rib, and fragmented elephant teeth belonging to at least two individuals were uncovered in Area C East, Layer 3. Most bones were very fragmented when exposed, thus requiring restoration [36].

Rabinovich and colleagues [36] hypothesized that the great number of large, unidentifiable, elephant bone chunks at the site might have resulted from two processes: breakage of long bones for marrow extraction [e.g., 40] and/or the production of tools [e.g., 41]. However, bone decomposition might have also occurred due to post-depositional processes.

Cut marks were observed on two ribs and a scapula of a straight-tusked elephant from Area B, suggesting that elephants were butchered at the site. Elephant bones shaped as tools were also found, including an elongated wedge-like tool and two bifaces [36]. Based on the lithic and faunal assemblages, and supported by the available relative and absolute chronology, the entire anthropogenic sequence was assigned to the Late Acheulean of the Levant [33, 36, 42].

Area C was divided into two sub-areas: C East and C West, located 8 m apart [43] (Fig 1D). In Area C West, covering 33 m^2^, five superimposed archaeological layers were exposed, labeled C1 to C5, from top to bottom [34]. Layer C3 in Area C West, the layer from which the items used in this study were retrieved, is the densest layer at the site, both in terms of flint items and animal bones [42] (Fig 1E).

Functional and residue analyses of flint items from Revadim have already provided exceptional results for the reconstruction of the activities performed at the site. Use-wear traces coupled with residues of fat, bone, and collagen fibers found on a conspicuous number of small flakes produced by means of lithic recycling testify to their utilization for precise cutting of animal carcasses at Layer C3 of Area C West [44, 45]. These results provide one of the earliest direct evidences for meat carcass processing and consumption by early humans in the Levant. Moreover, fat residues and use-wear traces were also found on a biface and a scraper, found in association with the remains of a butchered elephant at Layer B2 in Area B [37]. A study including 176 items from two different areas at the site (B and C) showed that flakes and tools were used equally in both areas, mostly to process soft animal materials, while evidence for the processing of wood and vegetal resources was also found [46].

## Materials and methods

The archeological context of Layer C3 was chosen due to the excellent preservation of residue and good potential for use-wear, as indicated in previous studies [44]. Moreover, we assumed that it would be best to focus our efforts on different lithic components from a specific archaeological context in order to reach a better understanding of the different human activities performed in Layer C3, rather than jumping from one context to another.

The methodology applied to the two samples analyzed in this study (chopping tools and flake cores) includes several different independent techniques: (1) use-wear analysis, (2) micro-residue analysis by means of optical microscopy, electron microscopy coupled with energy dispersive X-ray spectroscopy and Fourier transform infrared (FTIR) microspectroscopy and (3) a typo-technological analysis. Cross-checking the use-wear data with the residue results (conducted and double-checked through two independent spectroscopic techniques, FTIR and EDX) greatly increases the reliability of the functional interpretations. Previous use-wear and residue for lithic materials from Revadim and the Acheulo-Yabrudian Qesem Cave (Israel) have demonstrated the usefulness of the applied methodology and the likelihood of finding informative data in the context discussed here [44, 45, 47].

### Materials

Out of the great number of lithic artifacts found in the lithic assemblage of Layer C3 West at Revadim, a total of 53 chopping tools and 1,323 cores relevant for this study were classified [48].

While for the purpose of this study we analyzed the entire set of chopping tools retrieved from Layer C3 (S1 and S2 Figs in S1 File), we performed functional analyses for only a sample of flake cores (S3 Fig in S1 File). To select the core sample for this study, we randomly examined Layer C3 West cores and collected those made on pebbles until we obtained 50 such items. No permits were required for the described study, which complied with all relevant regulations.

Chopping tools are defined here as blanks with a central bifacially shaped ridge, often produced on pebbles [e.g., 3, 49, 50], following the original definition set by Leakey [19]. It is worth mentioning that unifacial chopping tools were not encountered in this assemblage.

Cores are differentiated here from chopping tools mainly by their morphology. Cores lack the central ridge, either bifacial or unifacial, characterizing copping tools. They do have one or more distinctive striking platforms. There is also a clear difference in terms of weight between the groups, with chopping tools being notably heavier, with an average of 100.3 grams for the chopping tools (median: 40 grams), compared to an average of 40.6 grams for the cores (median: 19.1 grams). A raw materials analysis comparing the flint types used for the production of the two types of artifacts is currently underway.

We selected 50 flake cores made on pebbles with one or two platforms. These are considered as flake cores, bearing at least one striking platform and one production surface, ubiquitous in the lithic assemblages of Revadim (S3 Fig in S1 File). We focused on cores made on flint pebbles, resembling the pebbles used for the shaping of chopping tools. It should be kept in mind that in contrast to the volumetric construction of a chopping tool with a bifacial ridge running at the midline cross-section of the pebble, and no distinct striking platforms or production surfaces, single platform cores are characterized by a clear distinction between these two surfaces, while their intersection is not located at the midline cross-section of the pebble. However, one-platform cores might resemble chopping tools in cases in which an acute angle is created by the intersection between the striking platform and the production surface. Two-platform cores differ significantly in shape and the use of volume from chopping tools.

**Fig 2:**
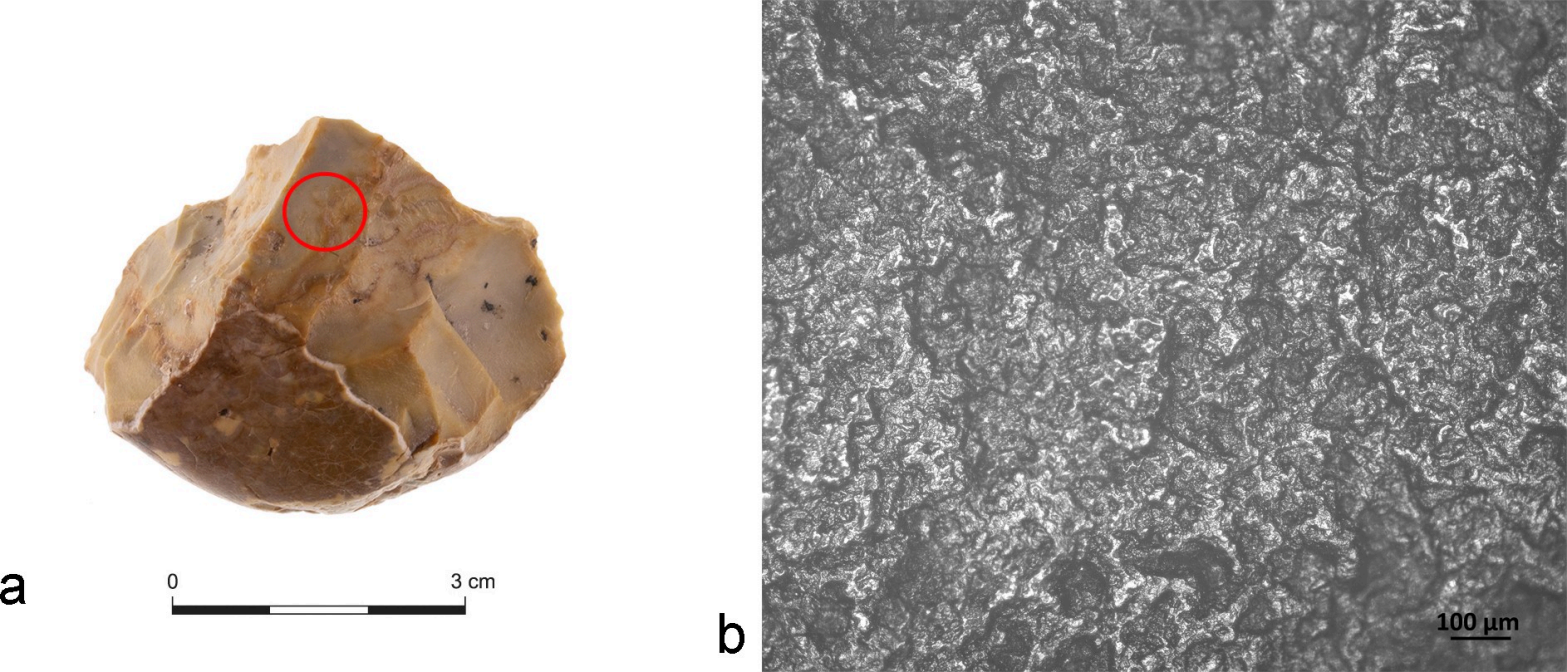
Post-depositional surface alteration identified in Area C Layer 3. a) A chopping tool with yellowish patination visible on the entire surface (red circle); b) Uniform and diffused sheen observed at high magnification on the surface of an artifact.

To the naked eye, the samples appear to be in relatively good condition, with complete outlines and relatively sharp ridges. At low magnification we see that the edges might have been damaged during excavation or by post-depositional weathering [33, 34]. Artifact surfaces appear with no particular pronounced smoothness or edge-scarring, suggesting that the items did not suffer strong mechanical stress due to post-depositional processes (except for one chopping tool). A geomorphological analysis in Area C showed that the burial of artefacts in Layer 3 was relatively rapid and caused by low energy water action [33, 34]. However, the presence of mostly alkaline water favored the chemical reaction responsible for patina formation. Indeed, artefact surfaces exhibited a change in their flint coloration towards brownish/yellowish shading (Fig 2A), while at the microscopic level, chopping tools exhibited a certain degree of alteration, resulting in highly reflective surfaces which mostly hampered polish microwear characterization (Fig 2B).

Despite of that, sedimentological and micromorphological analyses carried out at Revadim revealed that specific taphonomical conditions including the presence of mostly alkaline water, soil composition, the presence of heavy metal and carbonate, and soil pH, allowed the preservation of organic and inorganic residues, notably hydroxyapatite and adipocere [for more details on taphonomic processes affecting use-wear and residue preservation at Revadim see 44].

### Methods

#### Technological analysis

Chopping tools were divided into four types, as detailed below. Scars were counted, types of blank were indicated, and the presence of patina differences was recorded. The chopping tools were also measured (length, width and thickness) and were weighed. In addition, indications concerning the degree of homogeneity, texture and degree of translucency of the flint were provided. The cores from Revadim were classified by the number of striking platforms (1 platform, two platforms, or more than two striking platforms), as well as by the applied technological trajectory (prepared cores, flake cores, blade cores). In addition, indications were also provided in cases of cores with isolated removals, and for tested cores. For this study we used only flake cores, with one or two striking platforms.

#### Use-wear analysis

Use-wear analysis of the archeological and experimental tools was performed applying low- and high-power approaches [21, 22, 51–56]. Integrating the two methods allows the identification of use-wear traces at different magnifications. Macro and micro traces were described according to their morphological features. Edge damage was defined by the morphology of the edge scars along with their direction, location and distribution. Edge rounding was defined by its degree (low, medium, high).

Polishes were described according to their texture and topography along with their orientation, distribution and degree of rounding, while striations were defined by their morphology, orientation and location.

The archeological samples were first examined by the naked eye and with a Zeiss Discovery stereomicroscope with a zoom up to 8x, objective 1x, oculars 10x, and equipped with a LED ring-light at the Laboratory of Prehistoric Archaeology at Tel Aviv University. During this preliminary step, the artifacts were fully examined to assess the state of preservation and to identify and characterize edge damage and residues associated with use.

Subsequently, the artifacts were inspected with a Hirox RH-2000 digital microscope covering a magnification range of 35x-2500x at the Laboratory of Technological and Functional Analyses of Prehistoric Artefacts (LTFAPA) at Sapienza, University of Rome. At this stage the presence of morphological residues was noted, recorded, and later double-checked using spectroscopic techniques. After performing the residue analysis, the archaeological chopping tools and cores were observed under high magnification optical light microscopy (OLM) at the Tel Aviv laboratory using a Zeiss Axio Scope A1 with magnification ranging from 50x to 500x.

#### Residue analysis

Residues were first investigated morphologically. Residues identified on the archeological and experimental specimens were characterized and described at low and high magnification with the Hirox RH-2000 digital microscope and a Hitachi TM3000 scanning electronic microscope according to their appearance, consistency, color, inclusions, birefringence, and spatial pattern of distribution. The characterized residues were then subjected to two-step spectroscopic analysis using the micro-FTIR and SEM-EDX techniques.

The micro-FTIR technique was applied using a Bruker Optic Alpha-R portable interferometer with an external reflectance head covering a circular area of about 5 mm in diameter. The samples were placed directly in front of the objective, without preliminary treatments, and different spots were selected for analysis. The investigated spectral range was 7000–375 cm^−1^ with a resolution of 4 cm^−1^, and at least 250 scans were performed. Infrared measures were taken from at least three points from each specimen (proximal, medial, and distal) along the dorsal and ventral surfaces according to the used edge, and from at least two points on the inner dorsal and ventral surfaces of each item.

Fourier transform infrared spectroscopy proved to be an effective and reliable technique for in-situ residues characterization on archeological and experimental stone tools [57–60]. Despite of that, caution is needed when ancient residues on lithic tools are analyzed through FTIR. Infrared spectra of in-situ archeological residues generally suffer the low intensity of the bands of interest and the superimposition of signals produced by the stone reflection (Reststralhen bands) [57, 59, 61].

The energy dispersive X-ray analysis was performed on the chopping tools with a FEG-TESCAN-MIRA 3 scanning electron microscope coupled with an EDAX probe at the Department of Chemical Engineering Materials Environment, Sapienza, University of Rome. The X-ray analysis on one core was performed with a Hitachi TM3000 scanning electron microscope equipped with a SwiftED3000 probe at the LTFAPA Laboratory, Sapienza, University of Rome.

Various types of signals were used during the analysis of residues: secondary electrons (SE) at 5Kv mode were used to characterize topographic and textural traits while the back-scattered electrons (BSE) at 15 Kv mode provided elemental information through grey-scale images according to the atomic number. The energy dispersive X-ray spectroscopy was performed on each identified residue with two or three measurements taken on different spots of the same residue at 15 kV accelerating voltage in BSE mode with a magnification from 100x to 1500x and an acquisition time of 400 s.

The use of the two techniques allowed to strengthen the integrity of the residues while allowing the performance of the measurements directly on the artefact surfaces, without the need of any mechanical or chemical pretreatment of the sample. Moreover, the combined use of the two techniques, coupled with the morphological characterization of the residues, improves the reliability of the interpretation, which can be confirmed by cross checking the observations.

#### Cleaning procedures

Both the archaeological and the experimental specimens were observed before and after the cleaning procedures. The experimental specimens were quickly washed under water after experimental activity and before residue observation. Patches of residues were still firmly attached to the lithic surface after this procedure and were morphologically and chemically analyzed and recorded. In order to observe the microscopic use-wear, the replicas were placed in a chemical bath starting with diluted 3% acetic acid (CH3COOH) for 12 min, followed by diluted 3% sodium hydroxide (NaOH) for another 12 min. Finally, the objects were washed with deionized water in an ultrasonic tank for 5 minutes.

The archaeological specimens were first observed at low and high magnifications and later gently washed in deionized water prior to the use-wear and residue investigation. It should be mentioned that the finds were washed in water for cleaning purposes during fieldwork.

The specimens that underwent spectroscopic (micro-FTIR) and elemental analyses (SEM-EDX) were washed in deionized water in an ultrasonic tank for 5 minutes, allowed to dry, and then were sprayed with compressed air just before the analysis. For those specimens with fragile morphological organic residues such as fibers, chemical analyses were performed after a light wash by immersion in deionized water.

The artifacts were manually handled when excavated and sorted and were kept in plastic bags. After the limited manipulation following the excavation and the sorting procedures, the material was stored in separate plastic bags at the storehouse of the Israel Antiquities Authority and then at the Prehistoric Archaeology Laboratory at the Institute of Archaeology, Tel Aviv University. As previous studies indicated a good likelihood of identifying organic and inorganic residues on the lithic surfaces of artifacts from Area C, Layer 3 of Revadim [see 44], the typo-technological and morphometric analyses of the archaeological materials were performed only after the use-wear and residue analyses were completed. This was to prevent insofar as possible any modern contamination of the artifacts during the different stages of the analysis. According to our methodological protocol, the artifacts were always manipulated with powder-free sterile gloves during each analytical phase, and a plastic paraffin laboratory film (Parafilm^®^ M) was used to wrap the modeling clay (Blu-Tack^®^), supporting the pieces during the observations. We do wish to stress, however, that contamination from laboratory handling during the analysis cannot be avoided completely [see 62, 63]. Even with the use of latex gloves, invisible particles of dirt stay attached to the rubber glove and be transferred to on the artefact surfaces during laboratory manipulation. Thus, the importance of distinguishing non-use contaminant residues (such as micro skin flakes or micro modern dirty particles) [64] on the archaeological artifacts is paramount. Additionally, the integrated approach of different independent techniques presented here constitutes a valid and reliable method to successfully overcome the problem, providing reliable functional interpretations.

#### The experimental reference collection

Use-wear evidence and residues found on the archaeological specimens were compared to wear traces and residues observed on the replicas used during the chopping experiments. A comparison between archaeological and experimental use-wear and residue was also conducted [21–22, 44, 47, 58, 60, 62, 65–68].

The use-wear and residues results observed on the experimental replica are discussed in the S1 File, while a reference collection for chemical measurements using micro-FTIR and SEM-EDX of bone and other animal residues was prepared prior to this study. The results are available in 44, 47, while other comparisons can be found in the available literature [57–58, 62, 68, 69–74].

Replica of chopping tools were produced from unmodified pebbles, resembling the archeological chopping tools in size, shape and texture. These pebbles were collected at the vicinity of Revadim. Following the results obtained from the archaeological specimens (see below), the experimental items were used for bone processing, and specifically for chopping activities aimed at opening bones for marrow acquisition.

The experimental replicas were observed at the LTFAPA Laboratory with a Nikon Eclipse metallurgical microscope with magnification up to 500x. The replicas were examined on molds made with Provil Novo Light Fast Heraeus^®^ [see 75], while for the SEM analysis resin casts were made from these molds using the epoxy adhesive Araldite^®^ LY 554 plus hardener HY 956 [see 76].

### Experimental trials

After a preliminary observation of the archeological chopping tools, we noticed that most of the items exhibit a specific type of edge damage, along with some edge rounding, both associated with chopping activities. The animal residues observed are also associated with these activities. Thus, a series of experiments was conducted in order to test the hypothesis that the Revadim chopping tools were used to chop bone for marrow extraction, as proposed by Keeley, Semenov, Toth and others based on their replication experiments and the results of their microwear analyses of Lower Paleolithic chopper-cores [13, 21, 22, 27, 28, 30, 31, 77, 78].

The goal of the experiments was to test the efficiency of the chopping tool replicas in breaking open bones while creating microwear traces and residues that could be compared with the wear traces and residues observed on archeological chopping tools from Revadim.

Since the archeological chopping tools vary greatly in size, we replicated and tested small (weight < 50gr), medium (weigh < 120gr), and large tools (weight > 150gr). These were knapped from flint pebbles recovered from the site of Revadim and its vicinity by direct percussion, using a hard hammerstone (S3 and S4 Figs in the S1 File).

We performed seven experiments testing different combinations of variables: bones from different animals, different bone sizes, and different states of bone preservation to test the workability of the bones in different degrees of freshness. One chopping tool was also buried after use in order to record the morphological modification of residues after two months of interment (Table 1). We want to stress here that our experiments should not be regarded as a reconstruction of bone breakage activities conducted on-site but rather as an investigation into the applicability of chopping tools in breaking open bones. The inclusion of bones in different states of freshness and different sizes in the experimental reference collection allowed us to test and better understand the efficiency of chopping tools under different situations and conditions.

**Table 1:** List of the chopping tools used during the experimental activities of bone breakage with the main morphological and experimental variables.

Number	ID artefact	Weight (gr)	Edge angle	Activity	Worked material	State of preservation	Contact angle	Time	Efficiency
1	**Chopping tool#1**	172,8	85°	Chopping	Desiccated bone with periosteum (pork femur)	Dehydrated	90°	30’	Very good
2	**Cortical Flake#2**	/	25°	Removing periosteum (cutting)	Desiccated periosteum, bone, meat and collagen	Dehydrated	45°+90°	40’	Excellent
3	Chopping Tool#9 *buried	475	80°	Chopping (opening bone)	Bone without periosteum (cow femur)	Fresh	90°	20’	Very good
4	**Chopping Tool#10**	268	85°	Chopping (opening bone)	Bone with periosteum and meat	Fresh	90°	40’	Good
5	**Chopping Tool#2**	147	85°	Chopping (opening bone)	Bone without periosteum (pork femur, tibia, fibula)	Dehydrated	90°+45°	40’	Very good
6	**Chopping Tool#5**	188	70°	Chopping (opening bone)	Bone without periosteum (wild boar, 2 radii, ulna, tibia)	Fresh	90°	60’	Excellent
7	**Chopping Tool#7**	50	45°	Chopping (opening bone)	Bone (sheep)	Dry	90°	A few minutes	Inefficient

The experiments included chopping activities on fore and hind leg bones of small, medium and large animals (a roe deer, a pig, and a cow), since the marrow adipose tissue (MAT) is predominant in these skeleton parts [79]. Bones were processed in two conditions: 1) still covered by fat, muscular tissues, cartilage and tendons, and 2) after a cleaning procedure for removing meat, cartilage, and the periosteum membrane (S6 Fig in S1 File).

**Fig 3:**
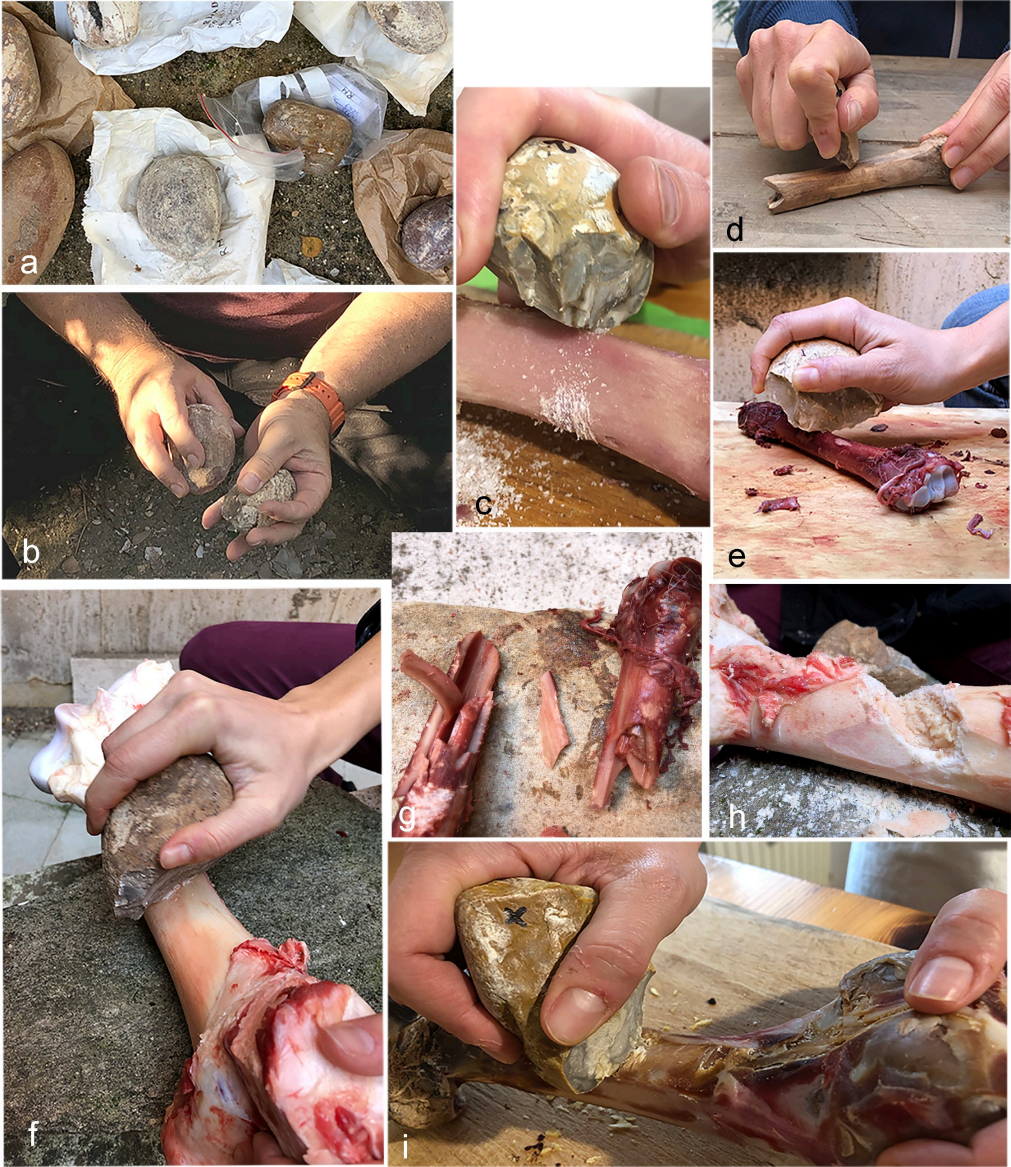
Representative images of controlled experiments carried out in order to create a reference collection. a) Pebbles of different sizes used to manufacture the experimental replicas; b) Chopping replica manufacturing process with a hard hammer; c) Experimental bone breaking activity (dehydrated pig bone without periosteum); d) Experimental bone breaking activity (dry sheep bone); e) Experimental bone breaking activity (fresh roe deer bone without periosteum); f) Experimental bone breaking activity (fresh cow bone without periosteum); g) Bone marrow accessed from a complete fracture; h) Bone marrow accessed from a large cut or fissure; i) Experimental bone breaking activity (dehydrated pig bone with periosteum).

We tested fresh bones, processed soon after butchery, dehydrated bones (retrieved from a raw ham, the Italian prosciutto crudo) that underwent an air-drying process assisted by ventilation under moisture-controlled conditions, and a completely desiccated bone (Fig 3C and 3I). A flat stone and a wooden support were chosen as an anvil in order to maintain the bones in balance during all the experiments; however, the chopping tools themselves did not come in contact with these supports during the activities.

Bone is generally considered a hard animal material, but hardness may vary depending on the state of preservation and the size of the individual bone. We noticed that fresh bones of large animals, such as femurs and tibias of bovine are thick, dense, elastic and hard to break. In contrast, the dehydrated pig bones were easier to break because of the aging process, mandatory for the production of raw ham. This process causes consistent water loss within the tissues (bone and meat lose up to 8% of their weight). This results in a thinner, softer, and less elastic bony matrix, but allows, at the same time, the preservation of edible marrow and a semi-dry and frayed consistency of the attached fleshy and connective tissues [see 80 for an overview of marrow preservation in bones; S5 and S6 Figs in S1 File]. This experiment provided us with a glimpse into similar situations of partial dehydration of animal tissues, which may occur under particular conditions of temperature, moisture, and ventilation in butchery localities (for example, when carcasses exposed to the open air are exploited only several days after the animal's death).

Concerning the workability of the bones, at least 60 strokes were needed to affect the dehydrated medium-sized pig bones, and more than 300 strokes all around the bone shaft circumference were required to break the bones in half to access the marrow (S5C Fig in S1 File).

Marrow was collected from several small, fresh bones of a roe deer after performing a few blows (S5A Fig in S1 File). Large fresh bovine bones were, in contrast, hard to break, and the marrow was accessed through a large, deep groove (S5B Fig in S1 File).

The complete dry bone could not be broken with our experimental tools (Fig 3D).

Clearly, successful bone breakage and the number of strokes required are influenced by several variables, including: 1) the applied force and pressure during the hit, and 2) the morphometry of the chopping tool in relation to the size and preservation conditions of the bone. The data reported here refer to experiments performed by a young female adult (F.V.) and an adult male, while for each experiment the most appropriate chopping tools for the type and size of the bones were chosen.

During the experiments, we noticed that it is difficult to chop through thick cylindrical bone shafts with tools that have steep and sharp cutting edges. It is easier in this case to cut it along its transverse axis. A series of several cuts are adequate to split bones of small to medium dimension but insufficient to open large and heavy bones in order to collect the marrow. In that case, tools such as shaped stone balls (polyhedrons/spheroids, usually much heavier than the chopping tools, often made of limestone and bearing massive ridges), which make contact with a large area of the bone, allow the force to spread over a large extent of the surface. This makes it possible to split large bones and extract the bone marrow in perfect condition [81].

As a rule, we noticed that it is preferable to remove fat, cartilage and periosteum membrane from the bone prior to the breakage attempt, especially if these materials are found in abundance. This facilitates bone breakage, as the adhesion between the stone cutting edge and the bone surface is optimal. As a general impression, we found the chopping tools efficient for chopping bone as long as their morphometric traits (weight, size, shape, and edge morphology) are suited to the physical characteristics of the worked materials.

Our observations and the results obtained from the microscopic examination of the chopping tools replica match the pioneering experimental works made by Keeley, Semenov and Toth [21, 22, 30, 31].

## Results

### Technological analysis of the chopping tools

**Fig 4:**
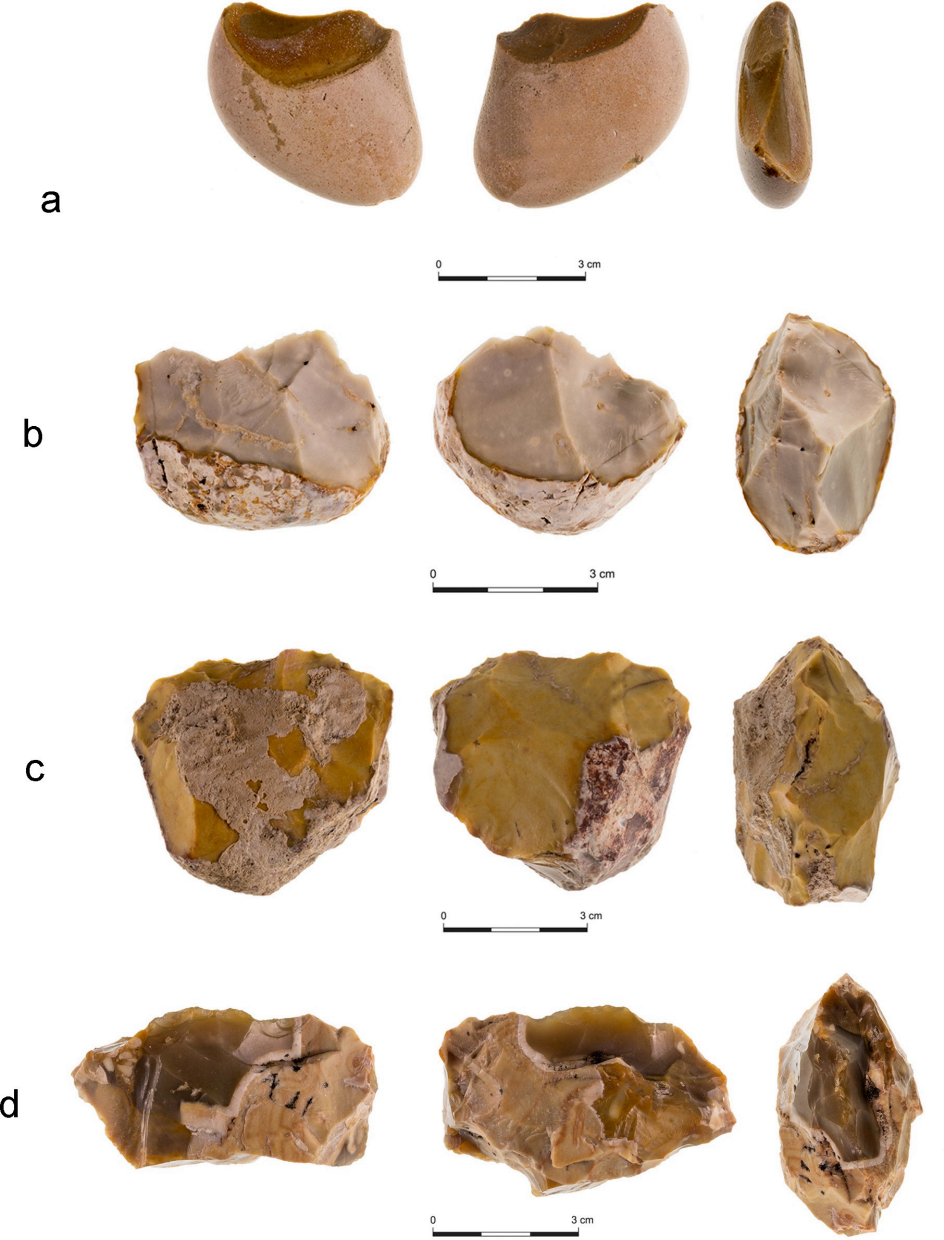
Classification of chopping tools from Area C Layer 3. a) Type 1; b) Type 2; c) Type 3; d) Type 4.

All chopping tools included in this study bear a bifacial central ridge. Most of the chopping tools (n = 48; 90.6%) are characterized by an unmodified base, still completely covered with cortex. The chopping tools were sub-divided into four types:

- Type 1 –chopping tools made on flat and thin pebbles, with partial bifacial knapping, only on the two sides of the ridge, with an unmodified base. Most of the cortex is still preserved. This group comprises almost half of the tools in the chopping category (n = 25; 47.2%) (Fig 4A).
- Type 2 –chopping tools made on thick rounded pebbles, with partial, more invasive bifacial knapping, mainly along the bifacial ridge. The bases are unmodified. Most of the cortex is still preserved (n = 16; 30.2%) (Fig 4B).
- Type 3 –chopping tools made on thick amorphous pebbles/nodules, with invasive bifacial knapping. Their bases are unmodified. Most of the cortex is still preserved (n = 9; 17.0%) (Fig 4C).
- Type 4 –Irregular chopping tools, which do not meet the criteria for any of the other types (n = 3; 5.7%) (Fig 4D).

**Fig 5:**
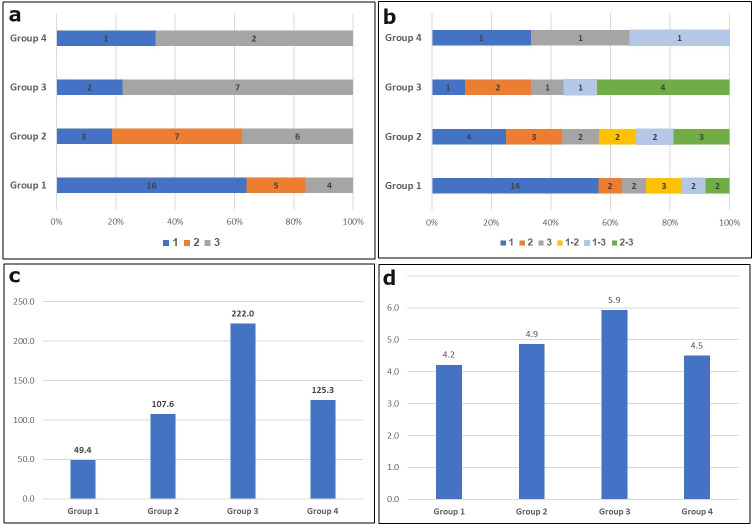
Data analysis of chopping tools. a) Frequencies of chopping tools by type and degree of flint homogeneity (1 = homogenous; 2 = fairly homogenous; 3 = heterogenous); b) Frequencies of chopping tools by type and flint texture (1 = fine; 2 = medium; 3 = coarse; 1–2 = fine to medium; 1–3 = fine to coarse; 2–3 = medium to coarse); c) Types of chopping tools by average weight (in grams); d) Types of chopping tools by average length (in cm).

The chopping tools can be further categorized into sub-types according to the traits of flint types from which they are made. Type 1 is dominated by homogenous fine-textured flint types (n = 16; 60.9%), while the flint types used in Types 2, 3 and 4 tends to be more heterogenous and coarser. Chopping tools from Type 1 are significantly lighter (47.4 grams on average; median: 32 grams) than those in the other types (146.25 grams on average, excluding Type 1; median: 60 grams). Chopping tools of Type 1 are also shorter (4.14 cm on average) (Fig 5).

Chopping tools are characterized by an average of 6.9 flake scars forming the bifacial ridge. Type 4 presents the highest average of scars (8.66 scars). The three other types are more consistent, with an average of between 6.38 and 6.67 scars per chopper (S1 Table in S1 File).

### Use wear analysis of chopping tools

Of the chopping tools, 41% (22 out of 53 specimens) have visible diagnostic microwear traces, while 19 (36%) do not. The 12 other tools (23%) had only weak generic unreliable use wear traces and were considered as non-diagnostic (Table 2)

**Table 2:** List of the archaeological chopping tools interpreted as used and related use-wear details.

Number	ID	L (mm)	W (mm)	T (mm)	W (gr)	Use-Wear description	Use-wear interpretation
1	#2 AP14c 71.13–08	40	55	43	114	**Edge damage**: compressions resulting in overlapped step-scars; **Edge rounding**: high	Chopping hard-material
2	#3 AS15c 71.14–12	46	36	34	50	**Edge damage**: crushed tip with overlapped step scars inside; step scars along the edge; **Edge rounding:** high **Edge damage prehensile area**: feather and hinge close-regular scars with transversal orientation	Chopping hard-material
3	#5 AV16a 71.03–01	4o	46	30	55	**Edge damage**: hinge close- regular scars; **Edge rounding:** medium	Chopping medium/hard-material
4	#8 AS15c 71.07–05	37	60	33	71	**Edge damage**: compressions resulting in overlapped step scars; **Edge rounding**: high	Chopping hard-material
5	#9 AR16d 71.05–07	63	56	43	158	**Edge damage**: hinge and step close-regular scars: **Edge rounding**: high	Chopping hard-material
6	#10 AR15d 71.18–17	37	44	21	37	**Edge damage**: compressions of the outer edge; **Edge rounding**: medium; **Polish texture**: rough to smooth; **Polish topography**: domed	Bone- smashing
7	#14 AX14a 71.11–09	45	50	15	53	**Edge damage**: small compressions of the outer edge and step transversal scars; **Edge rounding**: medium	Chopping medium/hard-material
8	#29 AU16b 71.02–00	58	65	27	149	**Edge damage**: crushing of the outer edge and step scars; **Edge rounding:** high	Chopping hard-material
9	#4 AS16c 71.04–02	50	39	14	33	**Edge damage**: feather/hinge close-regular and transversal scars; **Edge rounding:** low-medium	Scraping medium/hard material
10	#15 AS15a 71.15–07	31	37	24	24	**Edge damage**: overlapped steps close-regular scars; **Edge rounding:** medium	Scraping medium/hard material
11	#32 AR14b 71.13–11	25	29	12	9	**Edge damage**: feather and hinge close-regular scars; **Edge rounding**: medium	Scraping medium-hard material
12	#42 AR15b 71.16–06	32	35	16	21	**Edge damage**: feather close-regular **Edge rounding**: low	Cutting/incision on a medium material
13	#16 AR16a-d 71.03–70.90	32	51	11	26	**Edge damage**: scarring of the edge with overlapped step scars; **Edge rounding**: high	Chopping medium-hard material
14	#23 AP13c 71.14-	51	52	41	126	**Edge damage**: crushing of the outer edge and overlapped step scars; **Edge rounding**: medium	Chopping medium-hard material
15	#26 AW16a 71.03-70-97	52	65	55	255	**Edge damage**: crushing and compressions of the outer edge and overlapped step scars; **Edge rounding:** high	Chopping hard material
16	#28 AQ16b 71.08–03	94	89	52	664	**Edge damage**: crushed tip and step scars; **Edge rounding**: high	Chopping medium-hard material
17	#30 AS16d 71.27–23	58	66	44	136	**Edge damage**: crushed tip and feather scars: **Edge rounding**: high	Chopping hard material
18	#51 AS15 71.49–49	46	31	19	34	**Edge damage**: step close-regular scars; **Edge rounding**: medium	Chopping medium material
19	#52 AQ17c 71.03–70.01	35	53	20	44	**Edge damage**: overlapped step scars; **Edge rounding**: medium	Chopping medium-hard material
20	#12 AP13d 71.17–14	67	73	35	249	**Edge damage**: small overlapped step scars along the outer edge; **Edge rounding**: medium	Chopping unknown material
21	#13 AQ15c 71.35–34	60	52	15	66	**Edge damage**: feather and step wide-regular scars; **Edge rounding**: medium	Chopping unknown material
22	#18 AR18 71.12–00	53	42	16	47	**Edge damage**: / **Edge rounding: /**	Use is unknown (fractured)

**Fig 6:**
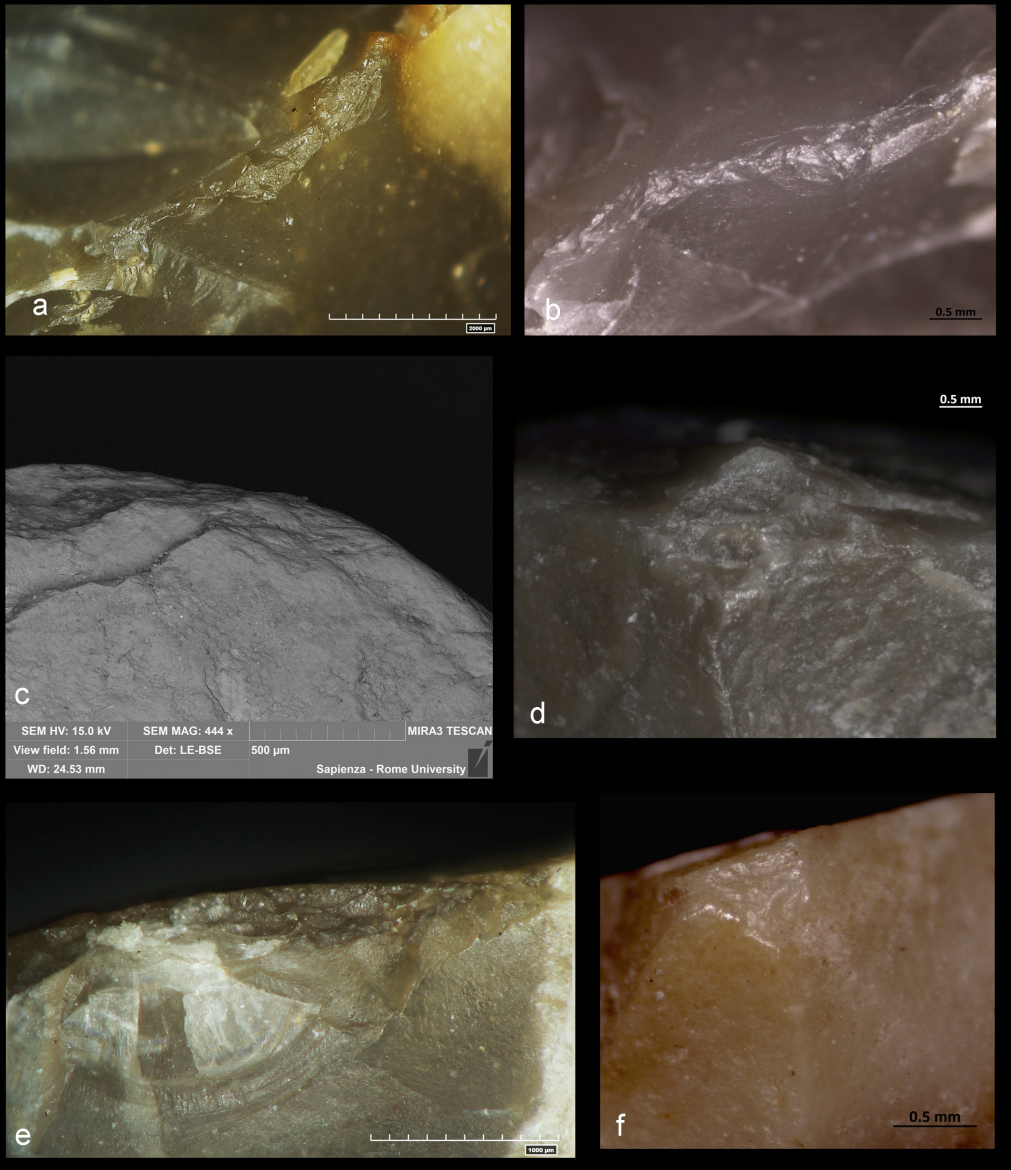
Overview of the edge damage observed on the archaeological chopping tools. a) Specimen #8 AS15c 71.07–71.05; b) Specimen #23 AP13c 71.14–71.13; c) Specimen #30 AS16d 71.27–71.23 (SEM image); d) Specimen #28 AQ16b 71.08–71.03; e) Specimen #8 AS15c 71.07–71.05; f) Specimen #30 AS16d 71.27–71.23.

Out of the 22 items with use-wear, 17 specimens were interpreted as having been used in chopping activities on medium to hard materials. Specific areas of the outer edges of these tools were worn, rounded, and scarred. Edge damage is represented by: 1) fracturing of the edge (Fig 6A and 6B**)**; 2) micro-crushing and rounding of the edge and/or edge asperities (Fig 6D and 6F); 3) micro-compressions of the outer edge appearing at high magnifications with pronounced rounding (Fig 6C); 4) overlapped deep hinge or step scars (Fig 6E). Edge damage distribution is not continuous along the entire profile of the bifacial ridge but is spotted where the tool impacted the worked material with the greatest force.

Three specimens exhibited traces related to scraping medium to hard materials. Edge damage is characterized mostly by step and hinge transversal scars (occasionally overlapped) distributed along the active edge. Edge rounding is always pronounced.

Only one chopping tool (CT#42 AR15b 71.16–06) in the entire sample was used to cut or grave medium material while a large fracture on the active edge of item CT#18 AR18 71.12–00 which probably occurred during use, may have removed the use wear traces.

**Fig 7:**
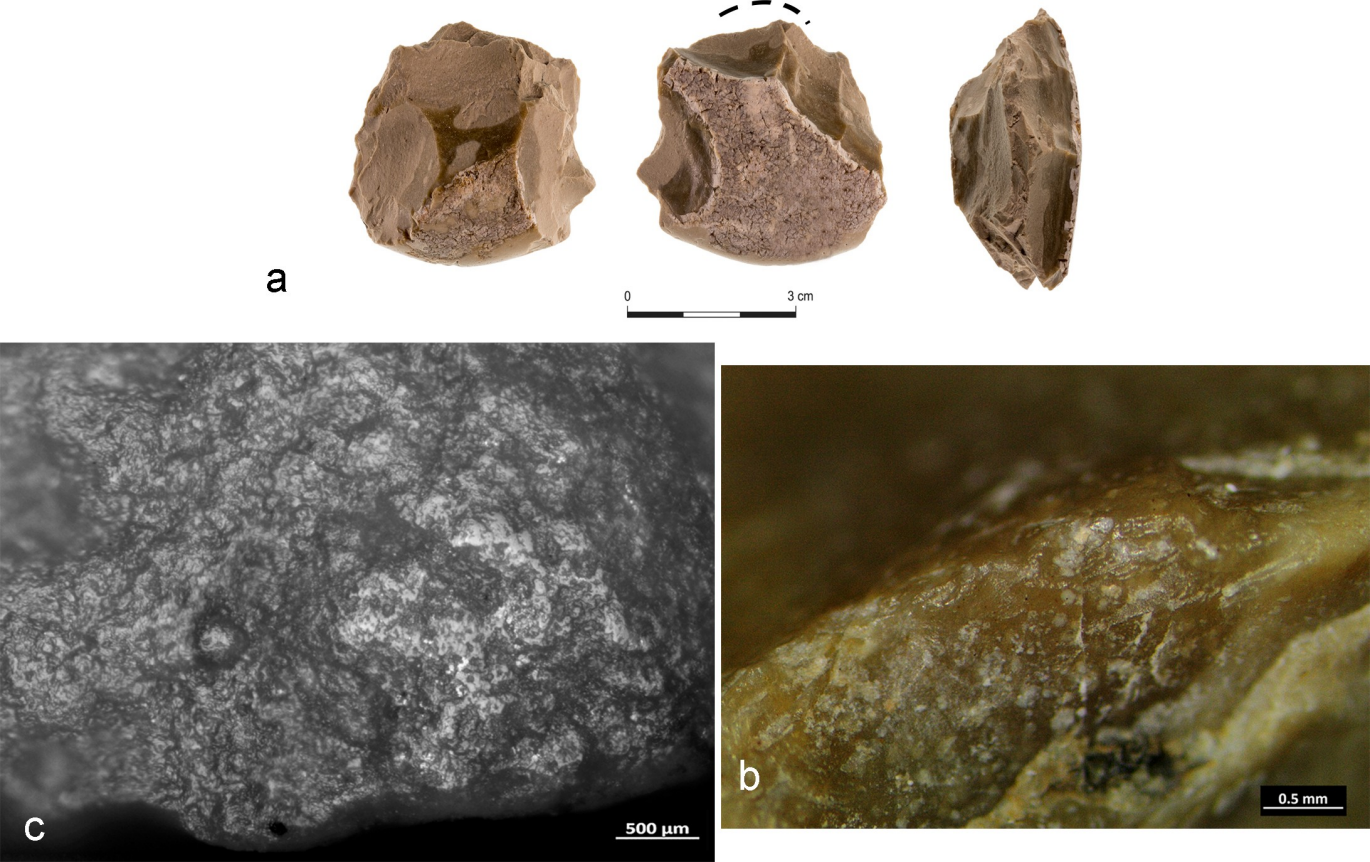
Archaeological chopping tool with related use-wear results. a) Specimen #10 AR15d 71.18–71.17; b) Edge damage; c) Bone-like polish observed on the functional edge. Dotted line indicates the functional area.

A detailed microscopic observation including polish characterization were possible on item CT#10 AR15d 71.18–17 showing better preserved lithic surfaces. A bright, domed, rough to smooth bone microwear was identified (Fig 7C).

**Fig 8:**
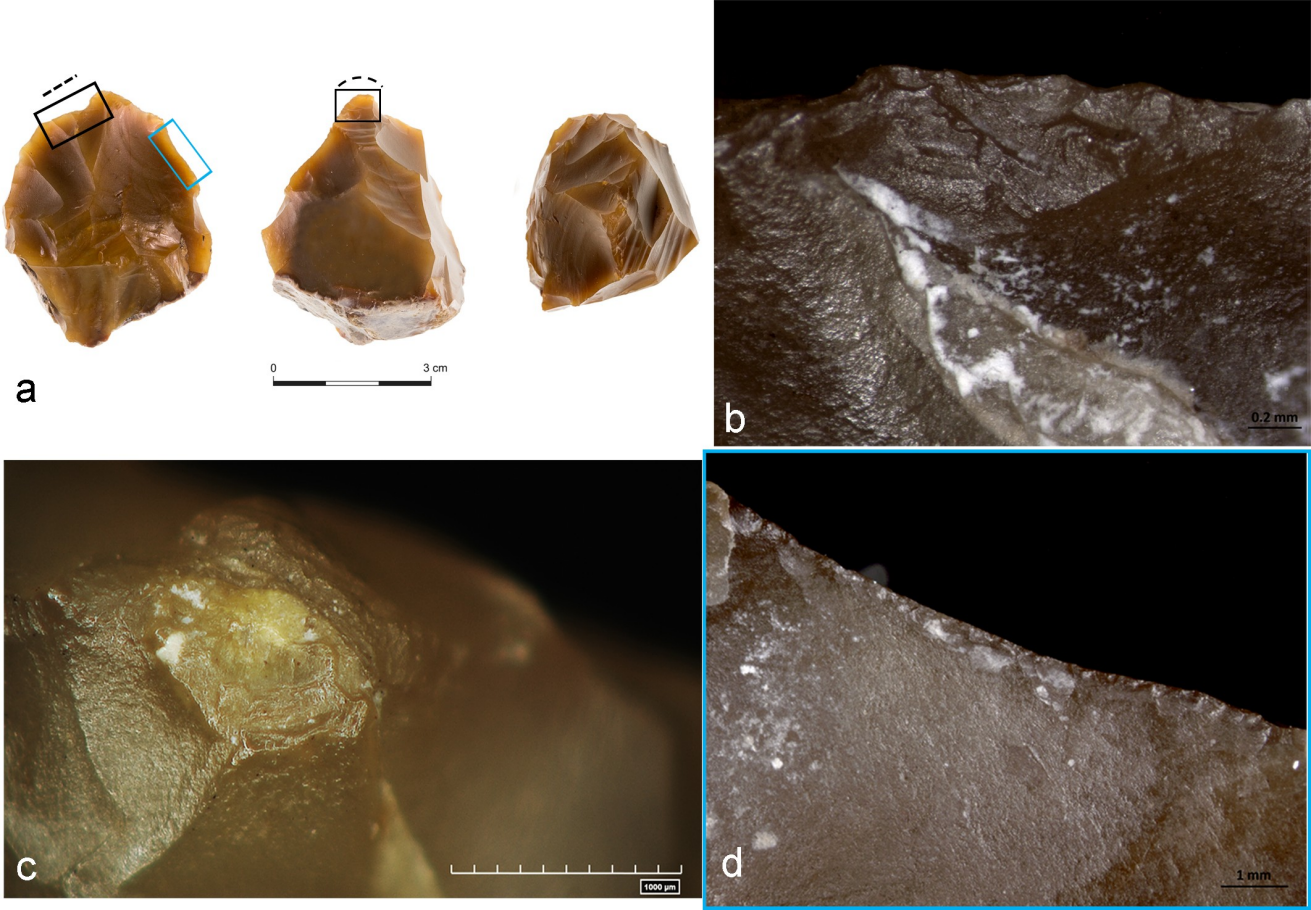
Archaeological chopping tool/core with related use-wear results. a) Specimen #3 AS15c 71.14–71.12; b) Edge damage c) Edge damage formed on top of a pointed area along the functional edge as a result of a battering activity on a hard material; d) Prehension wear. Dotted lines and black squares indicate the functional area, blue square indicates the prehensile area.

Our experiments showed that it is hard to reproduce and identify prehension traces on the parts of the chopper-cores that are covered with cortex. These are the areas that probably were held in hand when the tools were used. Prehension traces were only visible on item CT#3 AS15c 71.14–12, a borderline case that could be classified as a chopping tool or a core (Fig 8A). The almost complete absence of cortex allowed the identification of a series of overlapped tiny feather and hinge scars with a close regular distribution, that were interpreted as prehension traces (Fig 8D).

These prehension traces differ greatly from the compressions and scarring localized in the middle of the edge, but are consistent with a static pressure exerted in a marginal and localized spot of the chopping tool, outline by the finger during the manipulation of the tool in order to ensure a firm grip of the item [for examples and illustrations of prehension traces see, see 45, 56, 82].

### Residue analysis of chopping tools

**Fig 9:**
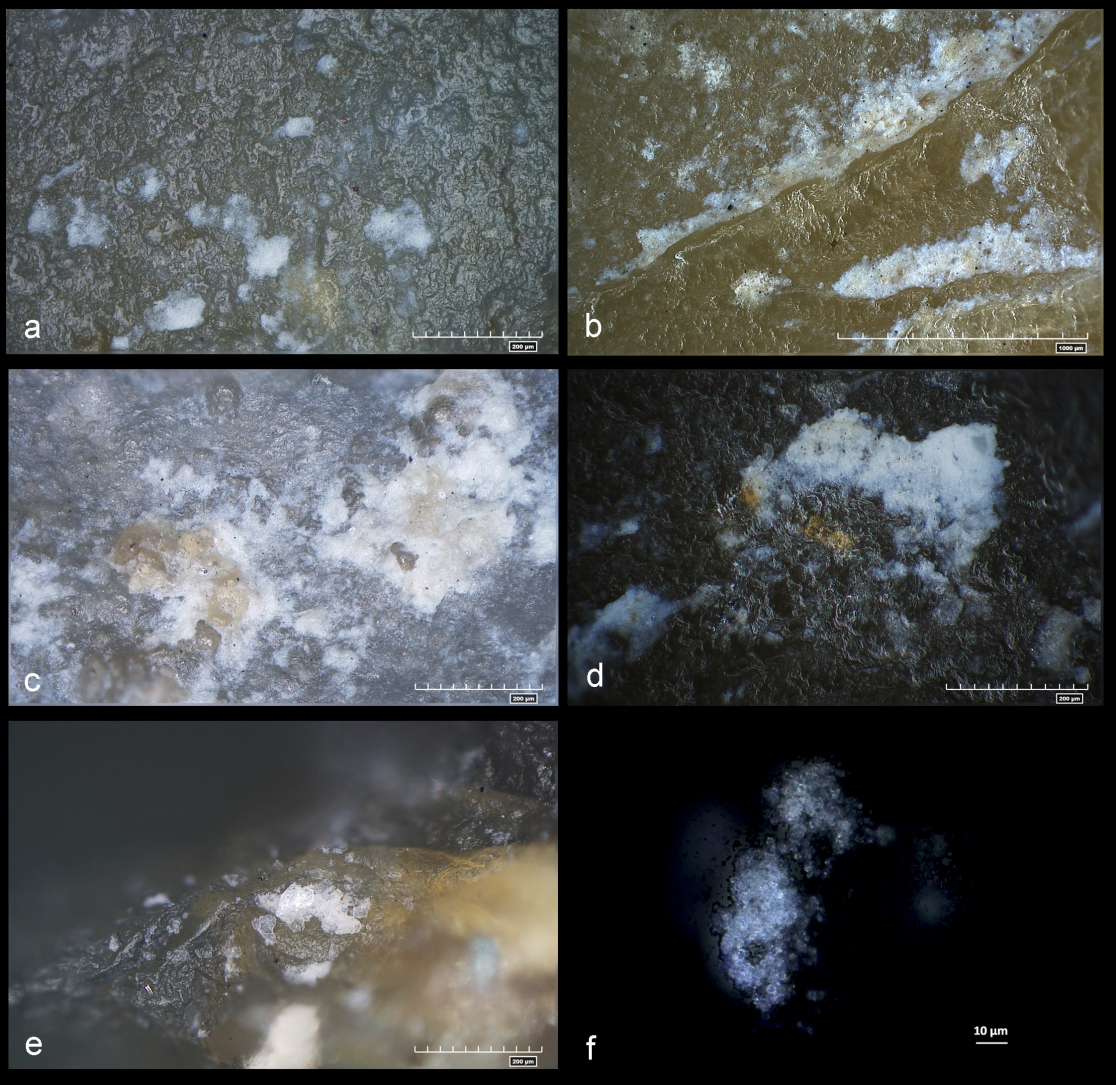
Archaeological micro-residues observed on the chopping tools. a) Whitish amorphous patches on specimen #4 AS16c 71.04–71.02; b) Whitish-yellowish amorphous patches on specimen #9 AR16d 71.05–71.07; c) Whitish amorphous patches with greasy yellowish inclusion on specimen #14 AW14a 71.11–71.09; d) Smeared whitish amorphous patches on specimen #3 AS15c 71.14–71.12; e) Crystalline white residues consistent with bony tissues entrapped in the use-edge scars on specimen #3 AS15c 71.14–71.12; f) Accumulation of superimposed bluish fat droplets along the active edge on specimen #3 AS15c 71.14–71.12 (cross-polarized light).

Morphological residues associated with the visible use-wear observed on the chopping tools were identified on 15 items (Table 3) and appear as whitish/yellowish amorphous patches displaying birefringence and transparency at high magnification. These residues exhibited, in some cases, yellowish amorphous or rounded structures embedded inside, with a glossy and greasy appearance (Fig 9A–9D). Crystalline white masses interpreted as bony tissues were also observed on one specimen along with a smeared mass of birefringent bluish fat droplets on the active edge of the same artefact (Fig 9E–9F). These residues fill the deep micro-depressions and the use-edge scars or they are smeared on the lithic surface just below the edge or several millimeters from it.

**Table 3:** List of the archaeological chopping tools interpreted as used and related residue description and interpretations.

Number	ID	Residue description	Micro-FTIR spectra absorption bands (cm^-1^)	FTIR interpretation	EDX elemental composition	EDX interpretation
1	#2 AP14c 71.13–08	Amorphous dense whitish-yellow deposit on the edge scar	~913 cm^-1^ P-O of hydroxyapatite	Bone	Calcium and phosphorus at proper ratio mineral part of bone (hydroxyapatite)	Bone
2	#3 AS15c 71.14–12	Amorphous dense white and yellow deposit smeared on the active edge consistent with bone residues. Birefringent bluish mass of fat droplets and white and translucent bone micro fragments	~2913/2848 cm^-1^ C-H of organic material; ~913 cm^-1^ P-O of hydroxyapatite; ~1573/1537 cm^-1^ C-O of fatty acid calcium salt carboxylate	Bone+adipocere	Calcium and phosphorus at proper ratio mineral part of bone (hydroxyapatite)	Bone
3	#5 AV16a 71.03–01	Amorphous white and yellow deposit along the active edge consistent with bone residues	~913 cm^-1^ P-O of hydroxyapatite	Bone	Calcium and phosphorus at proper ratio mineral part of bone (hydroxyapatite)	Bone
4	#8 AS15c 71.07–05	Amorphous white dense deposit along and below the edge consistent with bone residues	~913 cm^-1^ P-O of hydroxyapatite	Bone	Calcium and phosphorus at proper ratio mineral part of bone (hydroxyapatite)	Bone
5	#9 AR16d 71.05–07	Amorphous white dense deposit along and below the used edge consistent with bone residues	Not performed	/	Calcium and phosphorus at proper ratio mineral part of bone (hydroxyapatite)	Bone
6	#10 AR15d 71.18–17	Amorphous white dense deposit entrapped in use-wear scars below the edge and consistent with bone residues	~913 cm^-1^ P-O of hydroxyapatite	Bone	Calcium and phosphorus at proper ratio mineral part of bone (hydroxyapatite)	Bone
7	#14 AX14a 71.11–09	Amorphous dense and thick whitish/yellowish deposit below the edge consistent with bone residues	~913 cm^-1^ P-O of hydroxyapatite	Bone	Calcium and phosphorus at proper ratio mineral part of bone (hydroxyapatite)	Bone
8	#29 AU16b 71.02–00	White dense deposit along the edge	Not performed	/	Not performed	/
9	#4 AS16c 71.04–02	Patches of whitish deposit along the used edge consistent with bone residues	None	/	Calcium and phosphorus at proper ratio mineral part of bone (hydroxyapatite)	Bone
10	#15 AS15a 71.15–07	Abundant amorphous white deposit and whitish patches entrapped in flint scars along the edge and consistent with bone residues	None	/	Calcium and phosphorus at proper ratio mineral part of bone (hydroxyapatite)	Bone
11	#32 AR14b 71.13–11	Abundant white deposit and whitish patches entrapped in flint scars along the edge and consistent with bone residues	None	/	Calcium and phosphorus at proper ratio mineral part of bone (hydroxyapatite)	Bone
12	#42 AR15b 71.16–06	/	~1573/1537 cm^-1^ C-O of fatty acid calcium salt carboxylate	Adipocere	None	/
13	#16 AR16a-d 71.03–70.90	Small patch of whitish deposit along the used edge	Not performed	/	Not performed	/
14	#23 AP13c 71.14-	None	Not performed	/	None	/
15	#26 AW16a 71.03-70-97	Patches of whitish deposit along and below the edge	Not performed	/	Not performed	/
16	#28 AQ16b 71.08–03	None	Not performed	/	Not performed	/
17	#30 AS16d 71.27–23	Patches of whitish deposit along and below the edge consistent with bone residues	Not performed	/	Calcium and phosphorus at proper ratio mineral part of bone (hydroxyapatite)	Bone
18	#51 AS15 71.49–49	None	Not performed	/	Not performed	/
19	#52 AQ17c 71.03–70.01	Small patch of whitish deposit along the used edge	Not performed	/	Not performed	/
20	#12 AP13d 71.17–14	None	Not performed	/	Not performed	/
21	#13 AQ15c 71.35–34	None	Not performed	/	Not performed	/
22	#18 AR18 71.12–00	None	None	/	Calcium and phosphorus at proper ratio mineral part of bone (hydroxyapatite)	Bone

For a comprehensive characterization, the amorphous patches of residues were subjected to elemental and chemical analysis through SEM-EDX and micro-FTIR investigation (Table 3).

**Fig 10:**
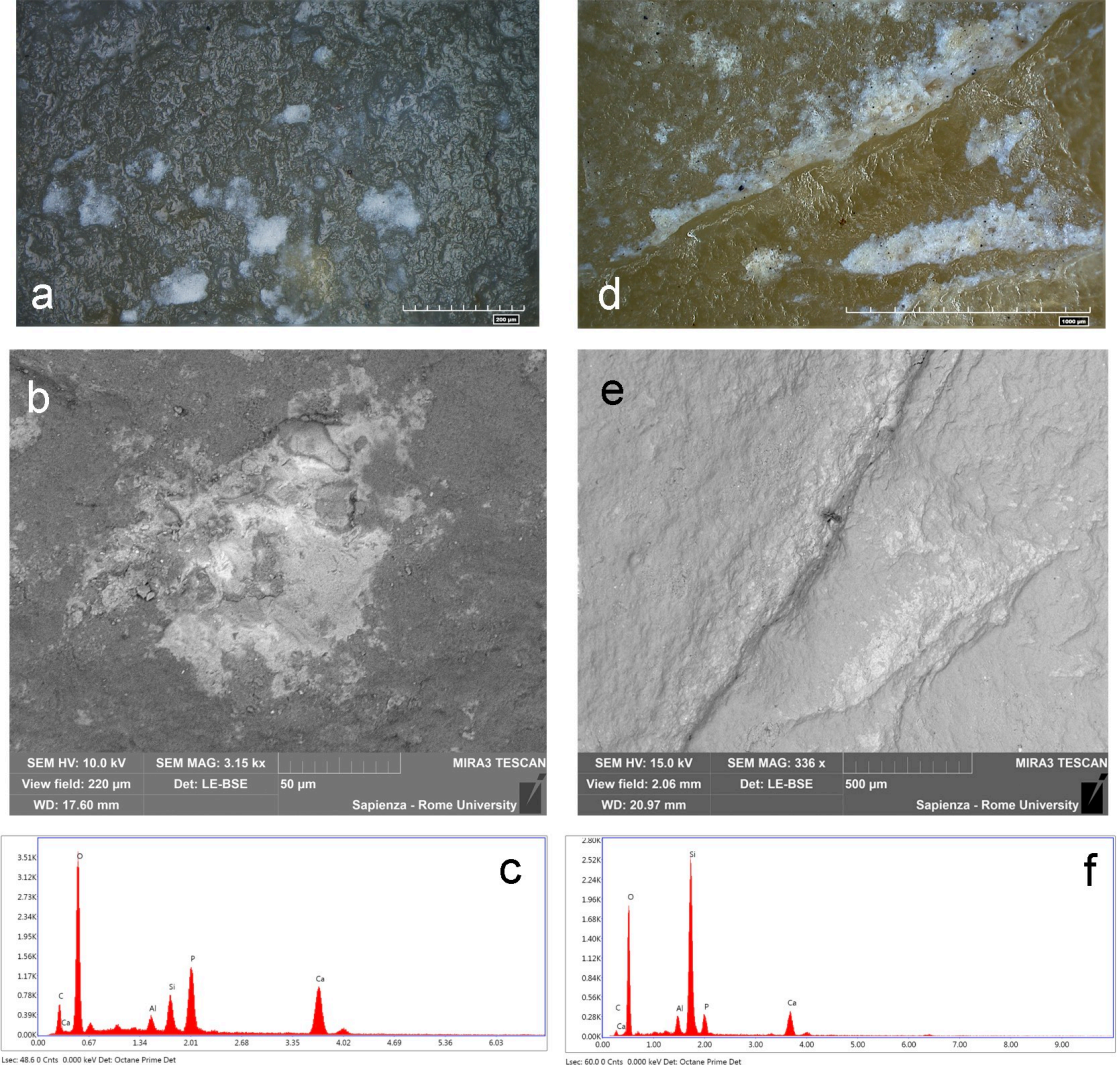
Morphological and elemental characterization of bone residues. a) Bone residues on specimen As16c 71.04–71.02; b) SEM micro-graph of the same residue; c) Elemental analysis; d) Bone residue in specimen #9AR16d 71.95–71.07; e) SEM micro-graph of the same residue; f) Elemental analysis. Intensity of the peaks varies according to the amount of residue analyzed. The Ca/P atomic ratio is equal to ~2.1.

The SEM-EDX analysis was performed on 14 tools. The whitish/yellowish patches of residues appeared at the scanning electron microscope with light and bright tonalities, standing out from the darker grey color of the flint substrate. They are compact/dense with a flat and regular topography (Fig 10B), but they also appear in powdery consistency. Residues are smeared in the micro-cavities of the lithic surfaces or embedded in tool’s scars (Fig 10E). The EDX measurement showed that residues are composed of major concentration of Ca, P and minor concentration of Al and Si (related to flint substrate) along with the stable C and O (Fig 10C and 10F).

By comparing the proportion between the detected elements, Ca and P always occurred at a stable ratio approximately equal to 2:1.

**Fig 11:**
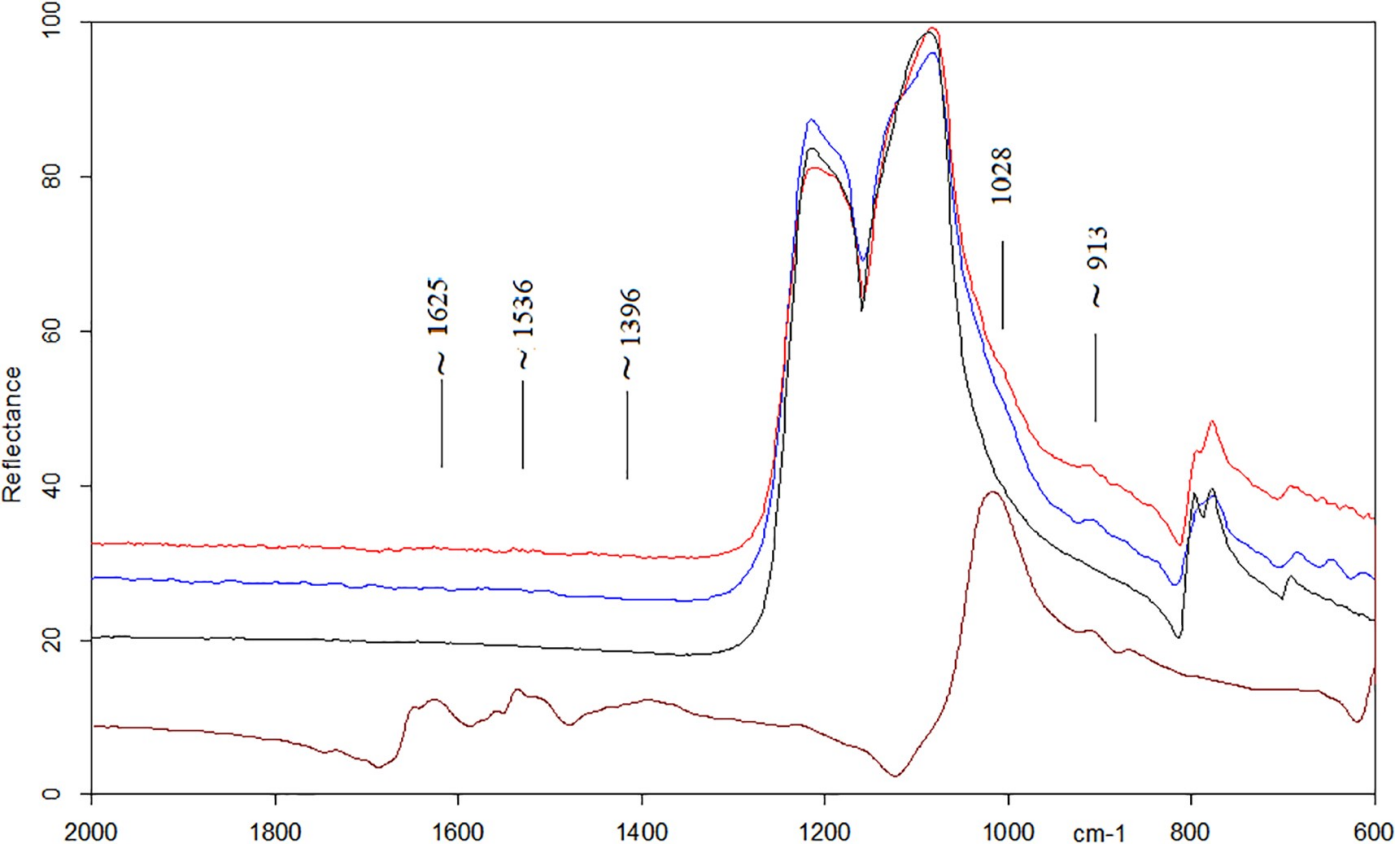
Comparison of micro-FTIR analysis of bone residues. Micro-FTIR performed on an experimental tool used for processing bone (red spectrum); micro-FTIR performed on the archeological chopping tool #8 AS15c 71.07–05 showing the peak at 913 cm^-1^ (blue spectrum), micro-FTIR performed on a spot without residues on the chopping tool #8 AS15c 71.07–05 (black spectrum); micro-FTIR performed on pure bone tissues (brown).

**Fig 12:**
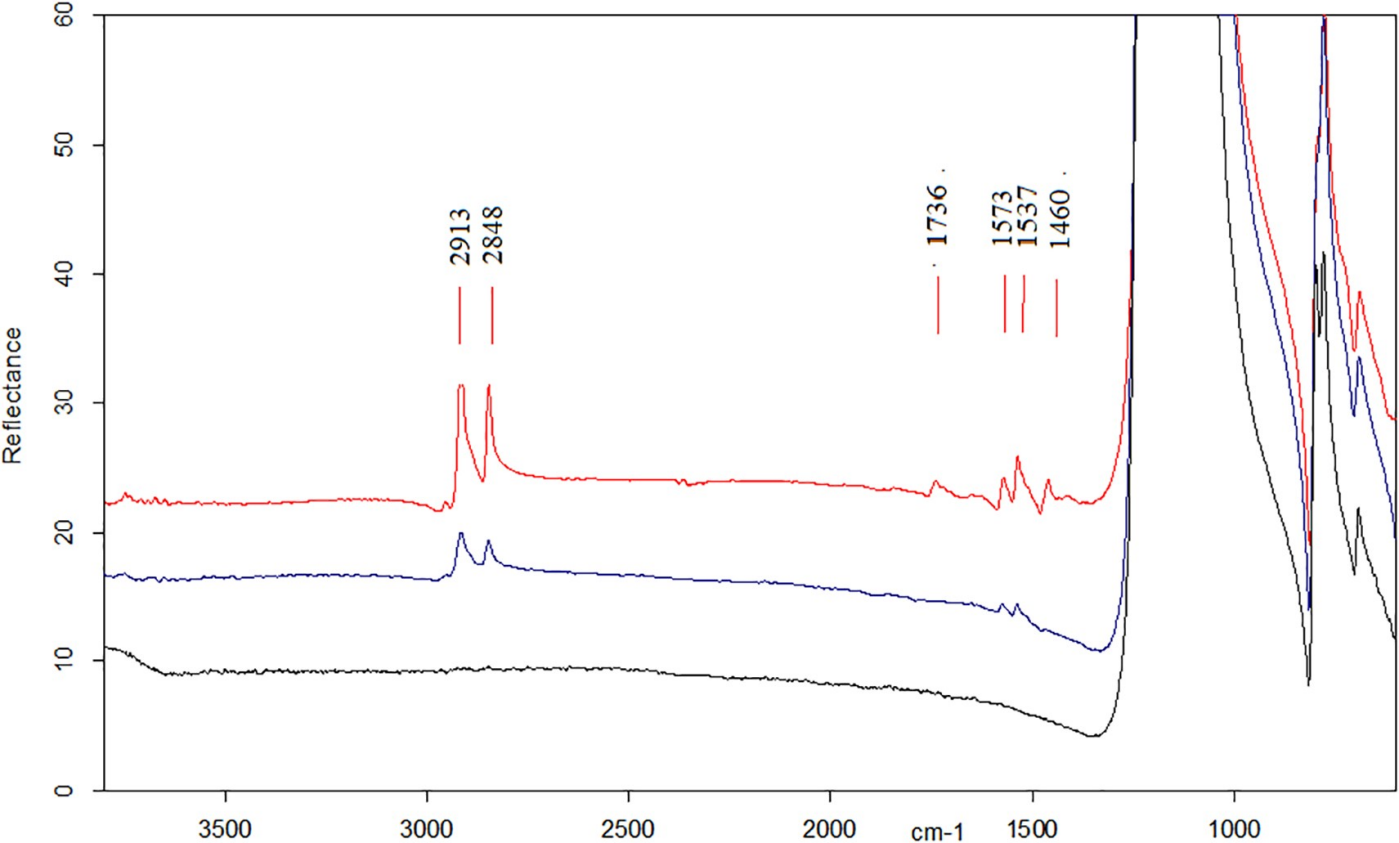
Comparison of micro-FTIR analysis of adipocere residues. Micro-FTIR performed on a tool few months after have been used for processing hide (red spectrum); micro-FTIR performed on the chopping tool #3 AS15c 71.14–71.12 (blue spectrum); micro-FTIR performed on a spot without residues on the chopping tool #3 AS15c 71.14–71.12 (black spectrum).

Micro-FTIR analysis was performed on 11 tools. All the micro-FTIR spectra show the very intense band at 1157 cm^−1^ (Si-O stretching mode) and less intense bands at 469 and 798 cm^−1^ (O-Si-O and O-Si-Al bending modes) characteristic of the cryptocrystalline silica (SiO_2_) which is the principal constituent of flint stones [83, 84]. Moreover, all peaks show an up- down reversal due to the *reststralhen* effect [57, 59, 85, 86]. Six out of eleven specimens reported the presence of a peak at 913 cm^-1^ suggesting the presence of hydroxyapatite (Fig 11 blue spectrum) [87]. Two tools displayed the presence of a doublet at the frequency of 1575/1536 cm^-1^ attributable to the stretching mode of fatty acid salts along with the C-H stretching vibrations of organic components detected in the range of 3000–2800 cm^-1^ (Fig 12 blue spectrum). These bands are in association with the peak at 913 cm^-1^ in one of the samples examined.

#### Interpretation of the residue results

The integration of three different levels of analysis for the investigation of residues (morphological, elemental and chemical) allowed to confidently interpret the residues as micro-remains of bone.

Bone consists in an organic component called collagen and an inorganic mineral component called hydroxyapatite with a chemical formula Ca_5_(PO_4_)_3_(OH). The major minerals in bone tissues are calcium and phosphorous and the most characteristic elemental signature of bone is the calcium-phosphorus (Ca/P) ratio. Stoichiometric hydroxyapatite has been reported with a Ca/P ratio of 2.15 while modern powdered cortical bone has a mean of 1.88 with a range of 1.61–2.02 [88]. The variation in the Ca/P ratio also depends on the bone types and the animal species analyzed [89].

Few archeological studies dealing with EDX measurements on residue characterization discuss the Ca/P atomic ratio of bone. Hayes and Rots [62] reported this ratio as Ca:P = 3:1 while Christensen [90] described the values equal to Ca:P = 2:1. In our EDX measurements, bone mineral Ca/P ratio always lies between 1.8 and 2.0 according to the weight percentage values.

Although bone residues show, in some cases, similarity with calcite accumulation when observed at the optical light microscopes (smeared patches of whitish accumulation, with an opaque aspect and transparent at high magnification), the two residues cannot be confused when a SEM-EDX analysis is performed. Calcite residues, which are also observed on Revadim samples and can be clearly interpreted as calcium accumulation, displayed a grainy topography under the SEM and their elemental composition is characterized by an overrepresentation of calcium in comparison to the other elements. Although less concentration of phosphorus might be found in calcite residues, the Ca/P was never recorded at the proper ratio (S7 Fig in S1 File).

The proposed interpretation for bone residues was further supported by FTIR analysis.

In the bone in-situ residues, the most intense peak of hydroxyapatite unfortunately lies around 1030 cm^-1^, overlapping the Si-O stretching mode of silica. However, the presence of bone produces a broadening at the lower frequency side of this mode and, in addition, a shoulder around 913 cm^-1^. The peak at 913 cm^-1^ is also observed on the spectrum of pure bone (Fig 11, brown spectrum).

Organic components of bone such as collagen and spectroscopically assigned to amide I, amide II, C-H scissoring vibration of CH_2_ and CH_3_, and Amide III [58, 71–73, 91] were observed only in the 1700–1300 cm^-1^ range of the bone sample spectrum. The presence of calcium and phosphorus in a proper ratio (~2:1) in all the specimens that also showed the IR peak at 913 cm^-1^ makes the spectroscopic assignments fully reliable.

For the two chopping tools showing the doublet 1573/1537 cm^-1^, we propose their assignment to adipocere, whose formation is due to the hydrolysis and hydrogenation of fatty tissues into a mixture of predominantly fatty acids as myristic, palmitic and stearic acids [92, 93]. The relative amount of these acids depends on the nature of the soil in which adipocere has formed [94]. Adipocere is a soap-like product containing the salts of mentioned acids, not soluble in water and able to invisibly fill the micro-holes of the microcrystalline of flint stone surviving for centuries. It should be noted that the doublet is present in the spectra of the mentioned acids and their salts along with other peaks [Fig 12 red spectrum, i.e. around 1740 cm^-1^ and in the 1500–1400 cm^-1^ interval, see 95], whose intensity decreases until disappearance when the amount of salts increase, leaving the doublet at 1573/1537 cm^-1^ as the most intense IR spectroscopic feature (Fig 12 blue spectrum) [96]. While for bone residues it was possible to cross-checking the FTIR results with their morphological and elemental characterization, the interpretation of adipocere only relies on the results of the infra-red analysis. Thus, more caution is needed in regards to its assignation, notwithstanding the good match between the experimental and the archeological results.

**Fig 13:**
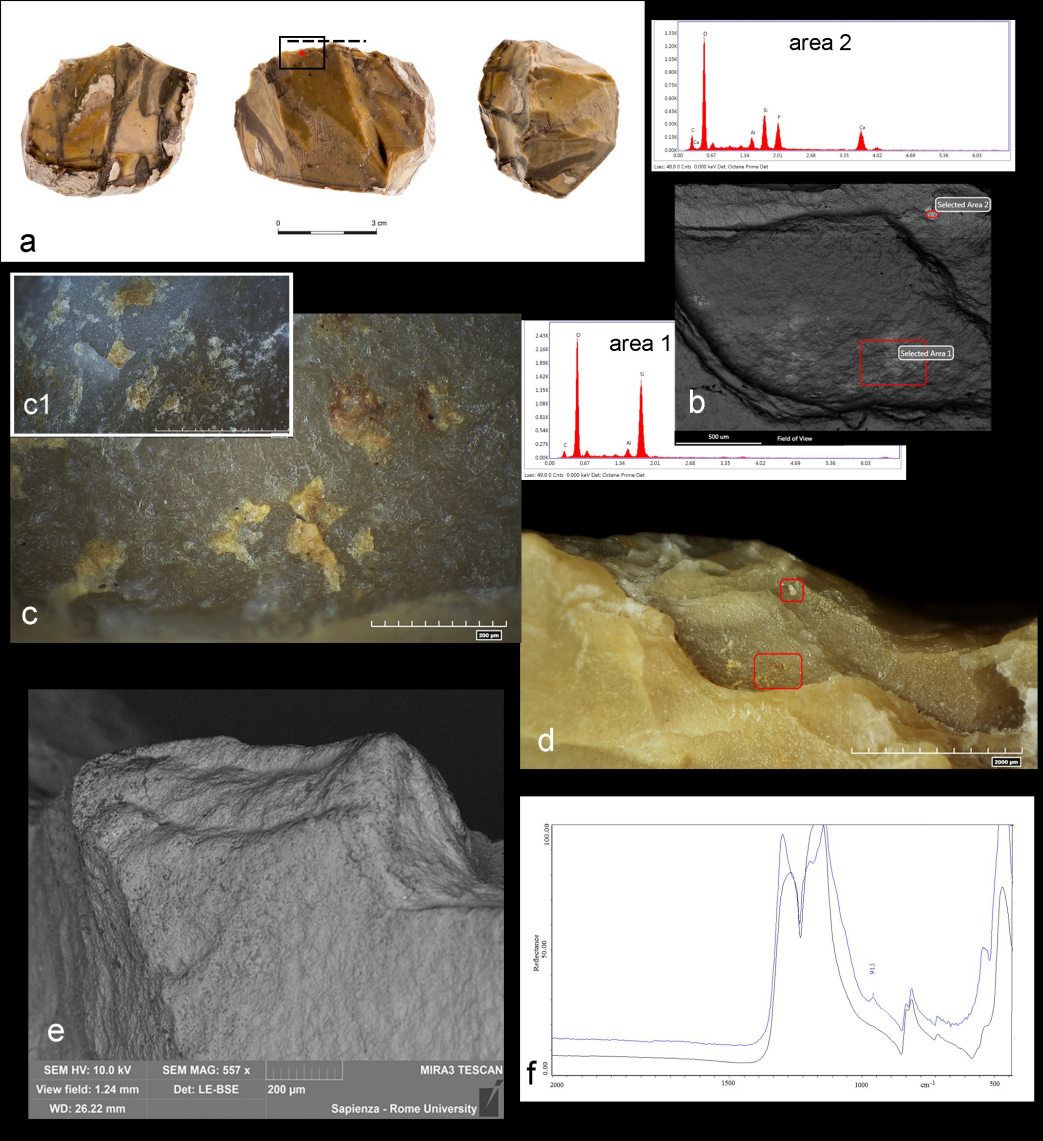
Archaeological chopping tool with related use-wear and residue results. a) Specimen #2 AP14c 71.13-71-08; b) SEM image of the yellow-brownish and white micro-residues encrusted in the scar along the functional edge and related SEM-EDX spectrum showing chemical composition of fat (area 1) and chemical composition of bone (area 2); c) Close-up of the yellow-brownish micro-residues and comparison with the experimental degraded fatty residues belong to the reference collection (c1); d) Location of the yellow-brownish and white micro-residues inside the scar along the functional edge; e) SEM image showing edge rounding related to the thrusting percussion activity; f) Micro-FTIR spectrum of bone observed in the area of residue (blue). Black spectrum shows a spot without residues. Red dot indicates the area of residue, dotted line and black square indicate the functional area.

The case of CT#2 AP14c 71.13–08, deserves a separate discussion. Yellow-brownish micro-particles, with a glossy and greasy appearance were found with micro-residues detected through both SEM-EDX and FTIR and interpreted as bone in a central edge scar (Fig 13B area 2 and Fig 13F).

Residues are embedded in the lithic surface and look dense and opaque but with a translucent aspect if imaged at high magnifications (Fig 10A–10D). Some of these particles appear whitish and gradually change their shades into yellowish coloration. Morphologically (i.e., in terms of appearance, consistency, color, opacity, and transparency), these micro residues show a striking resemblance to the micro-residues resulting from the biochemical degradation of marrow adipose tissue (MAT) in our burial experiment on item CT#9 (Fig 10C, C1). The topographical and elemental characterization by SEM-EDX analysis suggests an organic fatty origin for those residues, which was confirmed by the lack of additional compositional elements other than C, O, Si, Al. (Fig 10B, area 1). Fat tissues are basically composed of carbon and oxygen (the stable compound common in all organic and inorganic matters), along with hydrogen, where hydrogen is invisible to X-ray because of its atomic number (<5) [97]. As a consequence, the spectrum shows the compositional elements of fat (C, O) and of the rock substrate (C, O, Si, Al).

We exclude the possibility of interpreting the residues as modern contamination or sediment accumulation because they did no show the topographical traits of modern contaminants and because, if that had been the case, we would expect the residues to be composed of additional elements such as S, Na, Cl, Ka and Ca [64].

### Use wear and residue analysis of cores

Only four out of the 50 selected cores (8%) displayed traces related to use, mainly chopping and scraping activities on medium and hard materials. Edge damage is consistent with edge scarring and edge rounding found on the chopping tools samples (Table 4).

**Table 4:** List of the archaeological cores interpreted as used and related residue and use-wear interpretations.

Number	ID	Use-wear interpretation	Residue description	Micro-FTIR spectra absorption bands (cm^-1^)	FTIR interpretation	EDX elemental composition	EDX interpretation
1	#16 AR16d 71.08–04	Chopping hard material	None	Not performed	/	Not performed	/
2	#23 AQ15b 71.14–10	Chopping medium material	Long and short birefringent whitish and yellowish fibers. Some of them are perpendicularly oriented and blended in a whitish mass of white residue	~913 cm^-1^ P-O of hydroxyapatite 877 cm^-1^ δ_as_ CO_3_ of calcite ~1450 ν_as_ CO_3_ of calcite	Bone + calcium carbonate	Phosphorus, sulphur and calcium	Indeterminable
3	#35 AS15c 71.18–12	Scraping medium-hard material	None	Not performed	/	Not performed	/
4	#33 AS15b 71.11–06	Chopping	None	Not performed	/	Not performed	/

**Fig 14:**
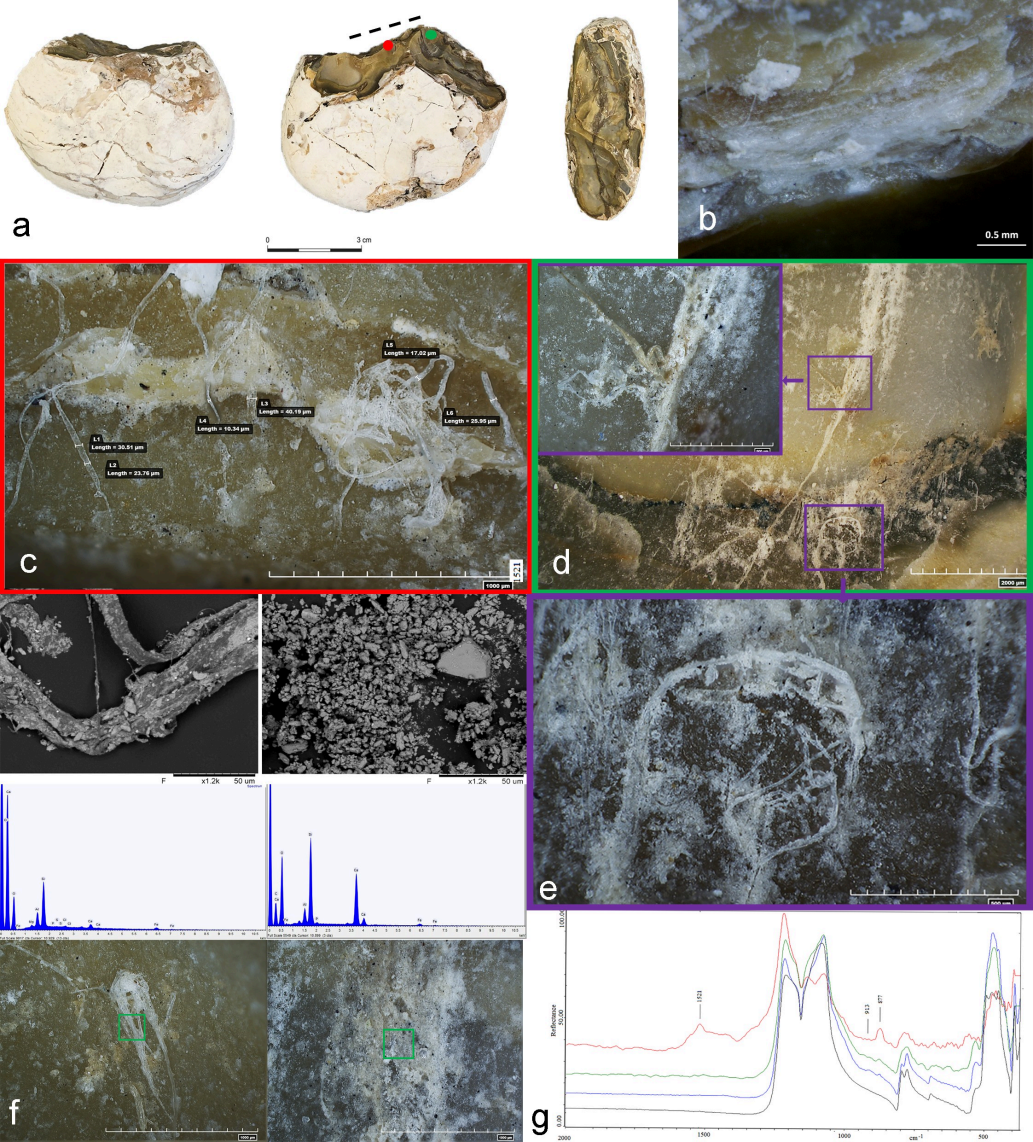
Archaeological core with related use-wear and residue results. a) Specimen #23 AQ15b 71.14–71.10; b) Edge damage; c) Group of fibers adhering along the utilized edge (red dot and square); d-e) Large group of fibers encrusted in a whitish mass of residue and close-up view of the twisted brownish long fiber; f) SEM-EDX spectra of a fiber and of the whitish residues with related SEM topographical view; g) Micro-FTIR spectrum showing the high absorption of calcium carbonate measured on the whitish residues (red) and micro-residue of bone detected along the edge and close to the area of fibers (blue and green). Black spectrum shows a spot without residues. Red and green dots show the location of fibers, dotted line indicates the functional area.

Morphological micro-residues were only observed on one core (#23 AQ15b 71.14–71.10), which resembles a chopping tool but without a clear central bifacial ridge at the intersection between the two faces of the pebble (Fig 14A). The used edge corresponds to the linear portion of its ridge, which displays overlapped step scars with a medium degree of rounding **(**Fig 14B). Along the used edge (Fig 14C, red square) and to a distance of few millimeters from it (Fig 14D, green square), we observed several groups of thin, long, round and flat fibers, transparent and birefringent, perpendicularly oriented, clumped together and firmly stuck on the lithic surface inside the micro-depression of the flint surface. Particularly noteworthy is a well-defined, twisted, long fiber, appearing flat and light brown in color, blended in a whitish mass of residue (grainy in texture), along with other groups of fibers with a clear transversal directionality (Fig 14D and 14E). Infrared analysis performed on the area of residues detected the presence of bone at 913 cm^-1^ and high presence of calcium carbonate (~877 and ~1450 cm^-1^ absorption bands), referring, in particular, to the white mass covering the fibers (Fig 14G). The stronger absorption at ~1521 cm^-1^ made it possible to classify the residue as sparitic calcite.

Due to its large dimensions, the specimen could not be placed in the SEM chamber to double-check the presence of bone micro-residue. We therefore decided to mechanically extract a sample of fibers and mount them on an aluminum stab covered by carbon film in order to observe their topographical characteristics. Fibers appeared at 5Kv mode both round and flat in shape and largely scattered by a veil of substance formed by geometrical small grains (Fig 14F). The fibers appeared dark grey in color while the grains are white; the color assignments were obtained in accordance with the atomic number using the secondary electron detector. The elemental analysis (EDX) of the fiber itself showed that it is composed of high concentrations of Si and Al along with low presence of Mg, P, S, Cl, Fe and Ca with the stable carbon-oxygen compound (Fig 14F). In contrast, measurements taken on the substance on top of them revealed high concentrations of Ca and Al and Si and a low percentage of P and Fe along with C and O (Fig 14F).

Based on these observations, we interpret the residue as ancient. Abundant concentrations of Si and Al were already noted as a constant in other measurements taken on residues from Revadim and were also recorded in measurements performed on a sample of bones retrieved from Layer C3. Thus, we believe that these two chemical elements—along with the high concentrations of calcium in this specific case—are associated with the taphonomical processes that affected the site during the burial period, although some small percentage of these three elements might be part of the fibers themselves.

That said, the information available so far does not allow us to specifically characterize the origin of the fibers. In the absence of reliable results from the spectroscopic analyses, the morphological characterization alone is not sufficient to confidently determine whether the fibers are of animal or vegetal origin. However, given the presence of bone detected by FTIR analysis, we prefer to interpret the core as having been used for chopping activity on a medium-to-hard material of animal origin as the more likely explanation.

## Discussion

The majority of the Revadim chopping tools follow the formal definition of these items (Types 1 and 2 in this study) as described by Movius [20] and Leakey [19]. These are usually oval in general shape, characterized by an unmodified base, with most of the cortex still preserved and a partial or invasive bifacially flaked single ridge resulting in a pointed or sinuous cutting-edge. A few other chopping tools, produced on thick amorphous nodules or flint blocks, were assigned to Type 3. Finally, Type 4 includes three artifacts that were produced on irregular nodules and do not correspond to any of the three other types.

Our view that these were designated, pre-planned tools rather than cores is supported by the partial exploitation of the pebble, as indicated by the average of seven scars per chopping tool along with the extensive cortical coverage. The low degree of reduction and exploitation supports a scenario in which the main goal was not flake production but rather the shaping of a sharp and solid bifacial ridge on a cortical pebble. The cortex cover might have been intentionally left intact to allow a firm grip while working with the tool [98–104]. The fact that many of these chopping tools were minimally flaked on one end of the pebble only, leaving most of the mass and volume of the pebble untouched, might further contradict the identification of these artifacts as cores. If flake production was the primary goal of removing flakes from a pebble, we would have expected more flakes to have been detached, as in the case of the pebble cores. What we see instead is only minimal modification of one end of the pebble, which was then discarded as such. Moreover, the bifacial shaping of the pebble is aimed at producing a robust and regular sharp edge in the case of the chopping tools, which is lacking in the group of the pebble cores. Notwithstanding the role of technological needs, the knapper's personal skills, and the availability of flint around the site which might have played a role in the intensity of the reduction, it is our contention that the morphology of these artifacts and their volume, combined with the use-wear results presented here and the small number of flakes removed from these chopping tools, all further demonstrate that most of these artifacts are in fact the intended tool, rather than cores.

The use-wear analysis coupled with the residue examination proved that the majority of chopping tools at Revadim Layer C3 were used in chopping activities involving the processing of medium and hard materials with direct evidence of bone processing (Table 5; S8 and S9 Figs in S1 File).

**Table 5:** Interpret function and material of chopping tools.

Worked Material	Number of items per activity				
	Chopping	Scraping	Cutting	Indeter.	Total
Bone	8	3			**11**
Medium material	1		1		**2**
Medium/hard	4				**4**
Hard	2				**2**
Indeter.	2			1	**3**
**Total**					**22**

Percussion damage and residue patterns demonstrate a direct and clear correspondence of most of the chopping tools with this type of activity. Although pounding activities on bone might also be performed using other types of tools, Layer C3 lacks other core-tool forms such as polyhedrons or spheroids useful for this type of activity [81], notwithstanding the fact that simple unmodified cobbles or hammerstones could have also been used as complementary items for this kind of task, as shown in our experimental trials (S10 Fig in S1 File). Chopping tools might have served as one component of a tool-kit oriented towards the extraction of bone marrow, used primarily as sharp-edged chopping tools aimed at fracturing the bone in order to facilitate and guide subsequent blows, conducted by a more massive percussor. In the case of the chopping tools, it seems that the bifacial ridge was the major element enabling efficient bone fracturing for marrow extraction. Unmodified stones lack such an element and thus do not enable an efficient and accurate execution of blows aimed at the breakage of bones. However, chopping tools, massive cobbles and hammerstones could have all been used in tandem to break long bones and expose marrow at Revadim.

By cross-checking the typo-technological data with the use-wear and residue interpretation, we observed that most of the chopping tools used to perform bone-smashing activities in Layer C3 are of medium dimension, measuring 5 cm in width on average and 50 grams in weight (n = 8), except for three larger and more massive items. The general morphology of their used-edge portions ranges from straight to semi-pointed, with steep or obtuse edge-angles ranging between 60 to 90 degrees. Our experiments showed that pointed extremities of the edge are preferable for percussive activities, while straight edges allow better control over the motion. The results of our experiments suggest a link between the morphometric features of the chopping tools and the physical characteristics of the worked material, which must have played a fundamental role in the successful outcome of the bone-chopping activity.

The three larger chopping tools (Type 3) weigh more than 100 grams and may have been used to process medium to large bones or large fragments of bones. According to ergonomic studies applied to archeological lithic artifacts, the size of the worked material is directly proportional to the size of the tool and, therefore, to the load/force applied by the tool [105–107]. As proof of this, longitudinal and transversal motions, which do not require a heavy loading force, were performed with the smallest (3 cm in length on average) and lightest (20 grams on average) chopping tools, among all those exhibiting use-wear traces. These data provide indirect evidence of the size of the animals processed using the chopping tools from Layer C3, in the absence of direct faunal analysis in this specific layer. The faunal assemblage unearthed in Revadim in different areas includes bovids, cervids, wild boar, equids, and elephants, among other species [33, 36]. Since chopping tools at Layer C3 range in size and weight, we can speculate that the more massive chopping tools were used to process medium to large bones of bovids or cervids (which are present at Revadim), but it seems rather unlikely that large bones such as those of elephants found in Layer C3 could have been processed with the chopping tools unearthed in this area.

Excluding one very large and heavy (600 grams) specimen interpreted as having been used in chopping a medium to hard material (possibly bone), the remaining specimens are of medium to small dimensions with rather short and limited cutting edges. Thus, they might have been used to break the bones of medium to small animals and/or used to treat parts or fragments of bones of large mammals, such as elephants. The very fragmented character of the bones in Layer C3 might support this interpretation, although taphonomical processes may have contributed to this fragmentation [36].

Moreover, the state of freshness of the worked material might have also affected the successful chopping of large bones. We verified that bones in a semi-fresh/dehydrated state of preservation are easier to break also when using tools lighter and smaller than the bone itself. That means that larger bones in a semi-fresh state could have been processed with chopping tools which do not necessarily exhibit suitable morphological characteristics.

Unfortunately, no specific patterns of residue and edge damage morphology or distribution were identified as a proxy to infer the timing of access to the carcass, discriminating between fresh and semi-fresh carcass processing. However, we cannot rule out the hypothesis that the Revadim hominins might have implemented both immediate- and delayed-return strategies on animal carcasses such as consumption on-site and drying meat and bone for later consumption, as suggested by Rabinovich and colleagues [36]. This can be explained by the need to cope with the consumption of large quantities of meat, hide, bone, and fats, as in the case of large animals such as elephants. Unbroken bone marrow can indeed persist in a fresh state for several days in medium to large bones without being subjected to microbial attack [108]. Supporting this scenario, to some extent, is the seemingly absence of upper and lower elephant limb bones at Revadim, which are the richest in terms of fat and nutrients (one should keep in mind, however, that these limb bones might have been the first to be fractured and thus are underrepresented as complete items but rather by the ubiquitous bone-chunks). Also, limb bones might have been removed and consumed elsewhere [36]. Moreover, the co-occurrence of cut marks and carnivore gnawing marks in Area B may indicate a complex scenario in which groups of early humans processed and consumed meat and fat at different times, while at other times some of the animal parts were also available for carnivores.

Delayed-return activities aimed at preserving bones for future marrow extraction is another feasible possibility, in light of the recently presented evidence from Qesem cave [80].

We would like to stress that, while further delayed butchery experiments coupled with cut-mark analysis in Layer C3 are required to shed light on the ongoing debate concerning hunting versus scavenging in the Acheulean, recent studies repeatedly support a scenario of active hunting performed by Paleolithic groups [109–111].

Our data also contribute to the discussion regarding another hotly debated yet unresolved topic: the function of chopping tools in Lower Paleolithic assemblages. Were these expressly produced to be used as tools, are they exploited cores aimed at the production of flakes or they should be considered as core-tools? Our results demonstrate that many of the chopping tools were used for targeted tasks, namely chopping hard materials, and specifically bone. In most cases, chopping tools exhibit minimal flaking (less than seven scars); the flake production potential of each pebble was clearly not fully exploited. We therefore believe that chopping tools were the desired end-products of this line of production, designed to fulfill a specific function. We cannot, however, exclude a scenario in which used and unused chopping tools also served as a source for flakes to be produced in times of need. As an example, item #52 AQ17c 71.03–70.01 exhibits patina differentiation in scar color and texture, testifying to its later exploitation as a core. Similar suggestions were also made by Zupancich and colleagues concerning a chopper found in Area B in Revadim [46]. Following the same reasoning, cores were also used, albeit rarely, for heavy-duty tasks when their morphology suited for the task at hand, thus emphasizing the grey zone between the two categories and the flexibility of core-tools form during the late Acheulean. Based on these findings we prefer to define the Revadim items as chopping tools instead of chopper-cores, notwithstanding their potential double function of being tools and cores at the same time.

## Conclusions

Combining different approaches and techniques, we present the first comprehensive set of results concerning the role of chopping tools in Late Acheulean Revadim. The rich lithic assemblage and the exceptional preservation of residues at Layer C3 provide important insights for the reconstruction of the activities carried out by the Revadim hominins. We have demonstrated that chopping tools were used to perform heavy-duty chopping/breaking tasks through percussion activities with direct evidence for bone processing, most probably for marrow acquisition. The fact that few such items were also used to cut and scrape animal matters reveals the probable multi-purpose potential of this category of tools, also reflected by their being a potential source for usable flakes (S11 Fig in S1 File). This also demonstrates the flexibility of the Revadim hominins in functional decision-making.

This study adds yet another piece to the puzzle of activities performed at Revadim, contributing to our knowledge of the different phases of animal carcass exploitation at the site. Thus far, the functional analysis of several lithic artifacts at the site proves that Revadim hominins were able to conceive a wide range of tools with distinct morphological characteristics (heavy- and light-duty tools) and employ them to obtain food (e.g., meat, fat, brain, marrow) and, perhaps, other useful materials from animal prey [37, 44–46].

These findings indicate that Revadim hominins could produce and use varied tool-types with appropriate functional characteristics, highlighting their resourcefulness in functional decision-making [37, 44, 46]. Moreover, the co-occurrence of simple forms such as chopping tools, along with more complex items in Layer C3 (i.e., bifaces), demonstrates the cultural variability and behavioral flexibility expressed by the Revadim toolmakers in conceiving their heavy-duty tool-kit. These findings shed new light on the cognitive adaptability and technological realm of Late Acheulean hominins in the Levant at the end of the Lower Paleolithic.

In a more general perspective, our results show the potential of studying the function of such ancient tools, also assisting researchers in reconstructing the role of chopping tools/choppers in Lower Paleolithic human adaptation strategies.

## Supporting information

S1 File (DOCX)
