# Supplementary material for: An integrated study discloses chopping tools use from Late Acheulean Revadim (Israel)
Source: PLoS One. 2021 Jan 19;16(1):e0245595. doi: 10.1371/journal.pone.0245595 (PMC7815122; doi:10.1371/journal.pone.0245595)
Supplement: S1 File — (DOCX) [file pone.0245595.s001.docx]

**S1 File.**

**An integrated study discloses chopping tools use from Late Acheulean Revadim (Israel).**

Flavia Venditti^1, 2*^, Aviad Agam,^3^ Jacopo Tirillò^4^, Stella Nunziante-Cesaro,^5^ Ran Barkai^3^

*^1^Department of Early Prehistory and Quaternary Ecology, University of Tübingen, Tübingen, Germany.*

*^2^Department of Classics, LTFAPA Laboratory, “Sapienza”, University of Rome, Italy.*

*^3^Institute of Archaeology, Tel Aviv University, Tel Aviv 69978, Israel*

*^4^Department of Chemical Engineering Materials Environment, University of Rome “Sapienza”, University of Rome, Italy.*

*^5^Scientific Methodologies Applied to Cultural Heritage (SMATCH), Italy*

*Corresponding author

E-mail: flavia.venditti@gmail.com (FV)


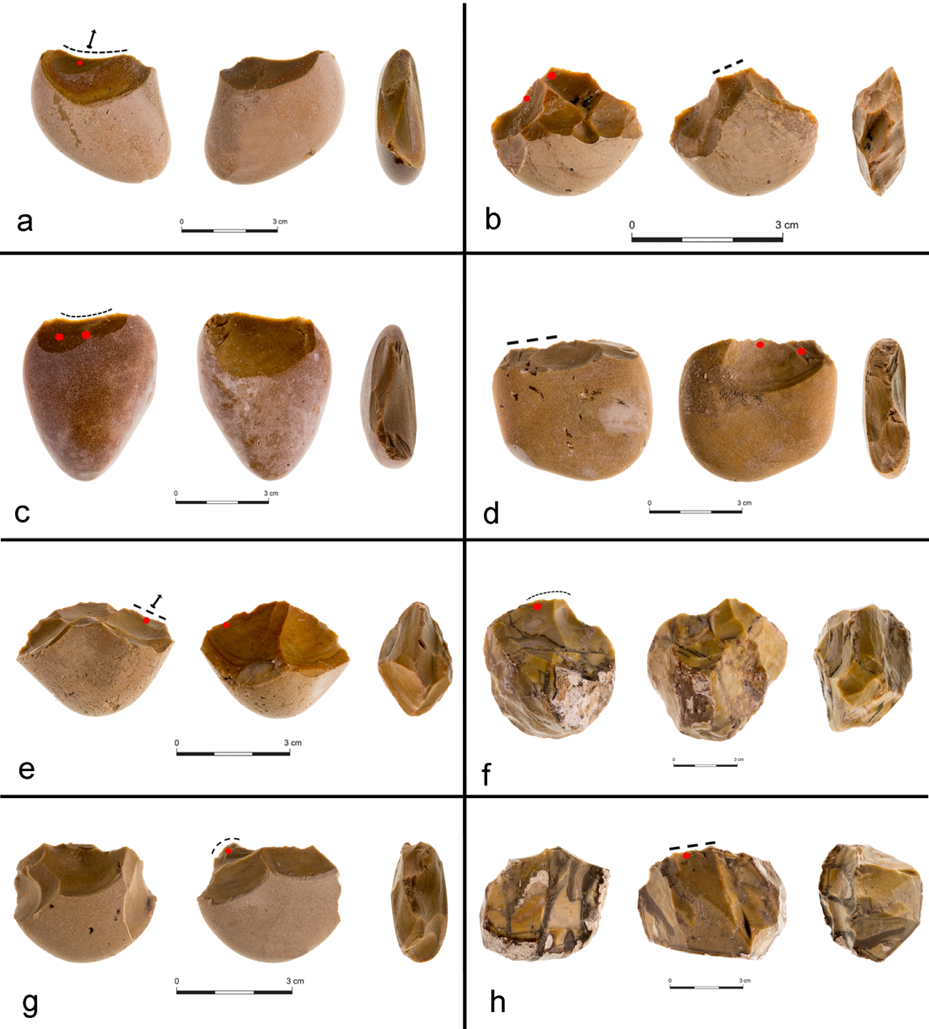


**Fig S1.** **A group of chopping tools retrieved from area C Layer 3.** a) Specimen #4 AS16c 71.04-71.02; b) Specimen #32 AR14b 71.13-71.11; c) Specimen #18 AR18 71.12-71.00; d) Specimen 14 AX14a 71.11-71-09; e) Specimen #15 AS15a 71.15-71.07; f) Specimen #9 AR16d 71.05-71.07; g) Specimen #42 AR15b 71.16-71.06; h) Specimen #2 AP14c 71.13-71.08. Red dots indicate the area of residue, dotted lines indicate the functional area.


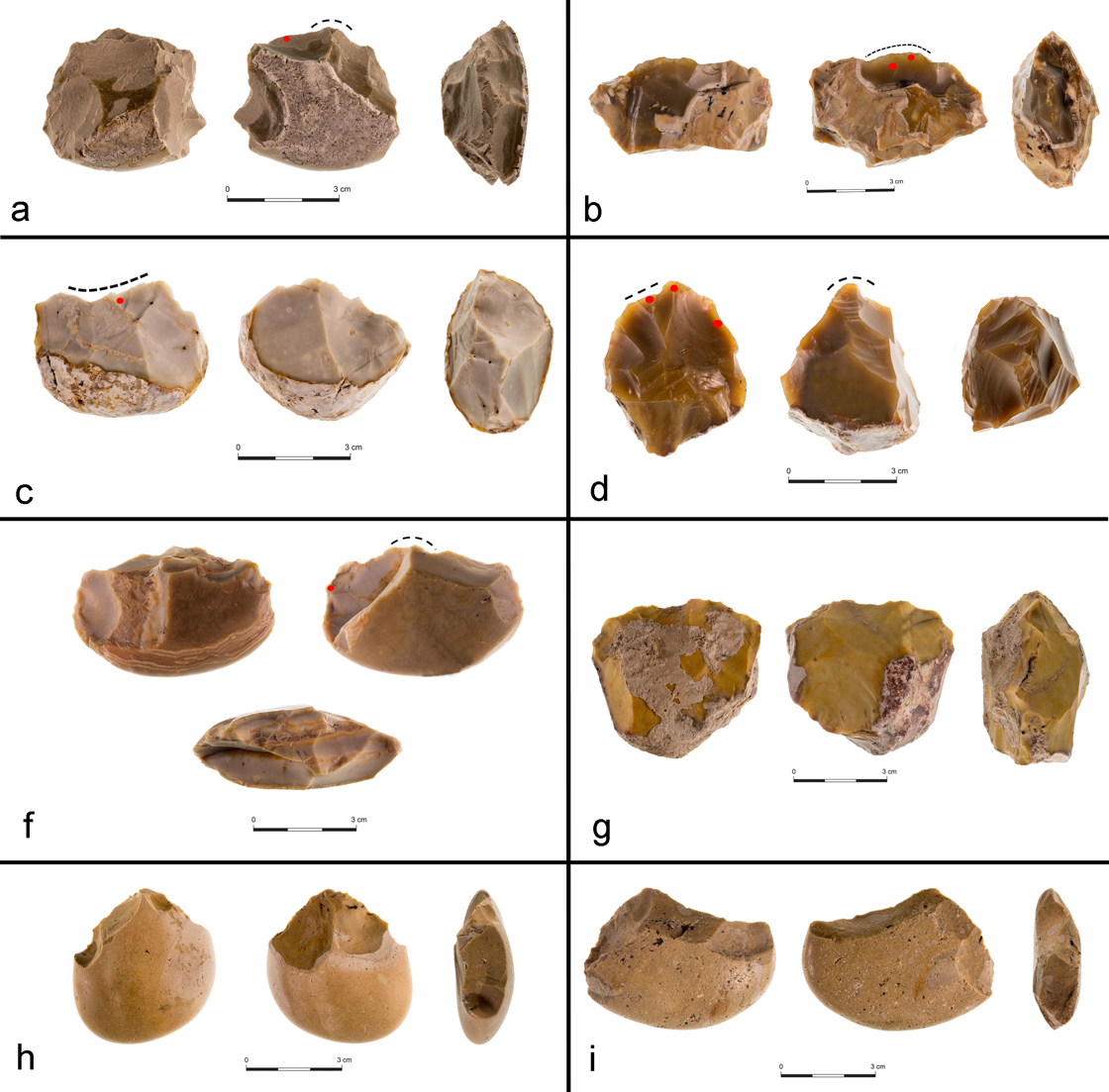


**Fig S2**. **A group of chopping tools retrieved from area C Layer 3.** a) Specimen #10 AR15d 71.18-71.17; b) Specimen #8 AS15c 71.07-71.05; c) Specimen #5 AV16a 71.03-71.01; d) Specimen #3 AS15c 71.14-71.12; e) Specimen #52 AQ17c 71.03-70.01; g) Specimen #21 AW15a 71.17-71.15; h) Specimen #1 AP14a 71.14-71.11; i) Specimen #6 AS16c 71.10-71.03. Specimens g), h), i) were interpreted as unused. Red dots indicate the area of residue, dotted lines indicate the functional area.


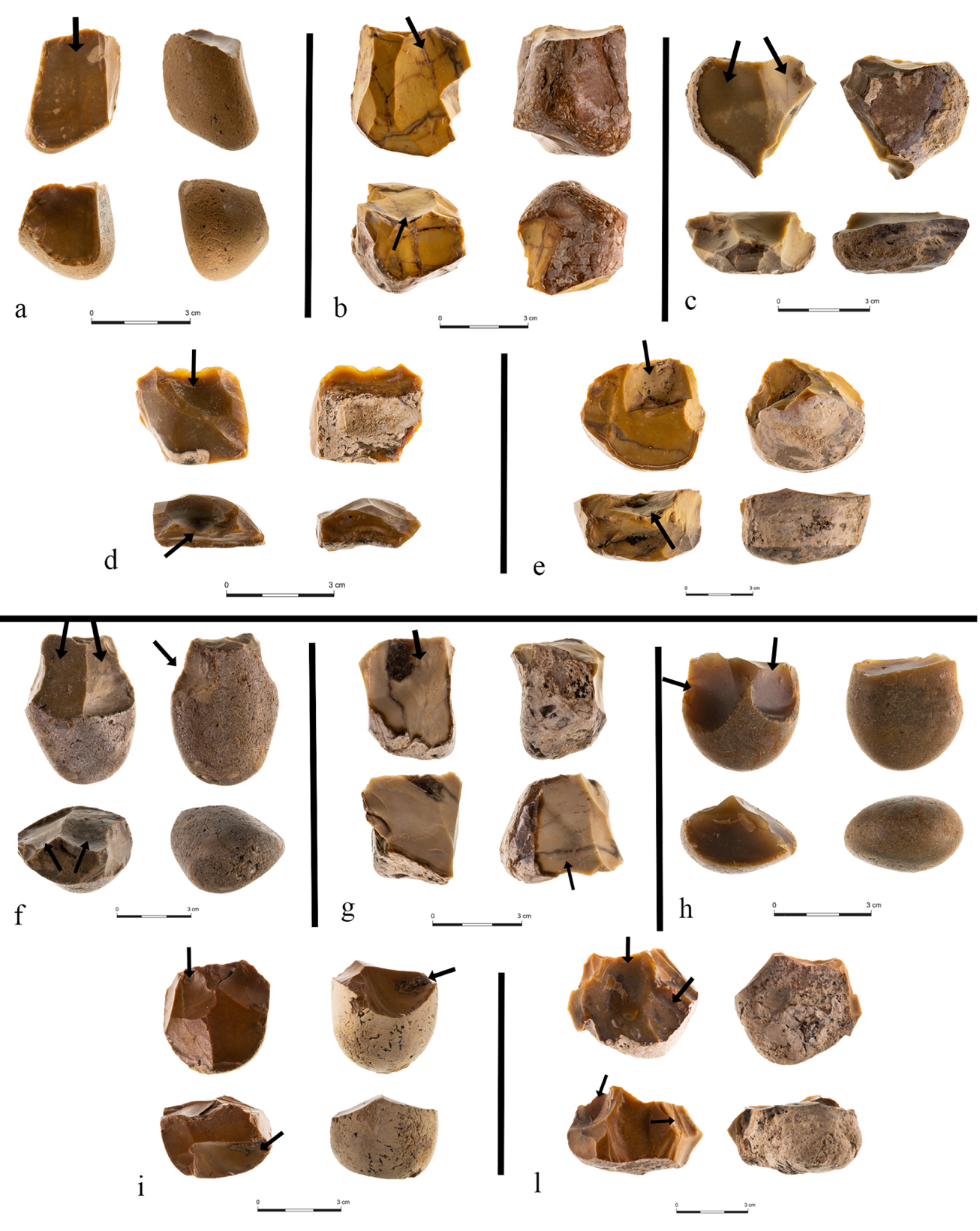


**Fig S3: Cores retrieved from area C Layer 3.** a-e) One striking platform cores; f-l) Two striking platform cores.


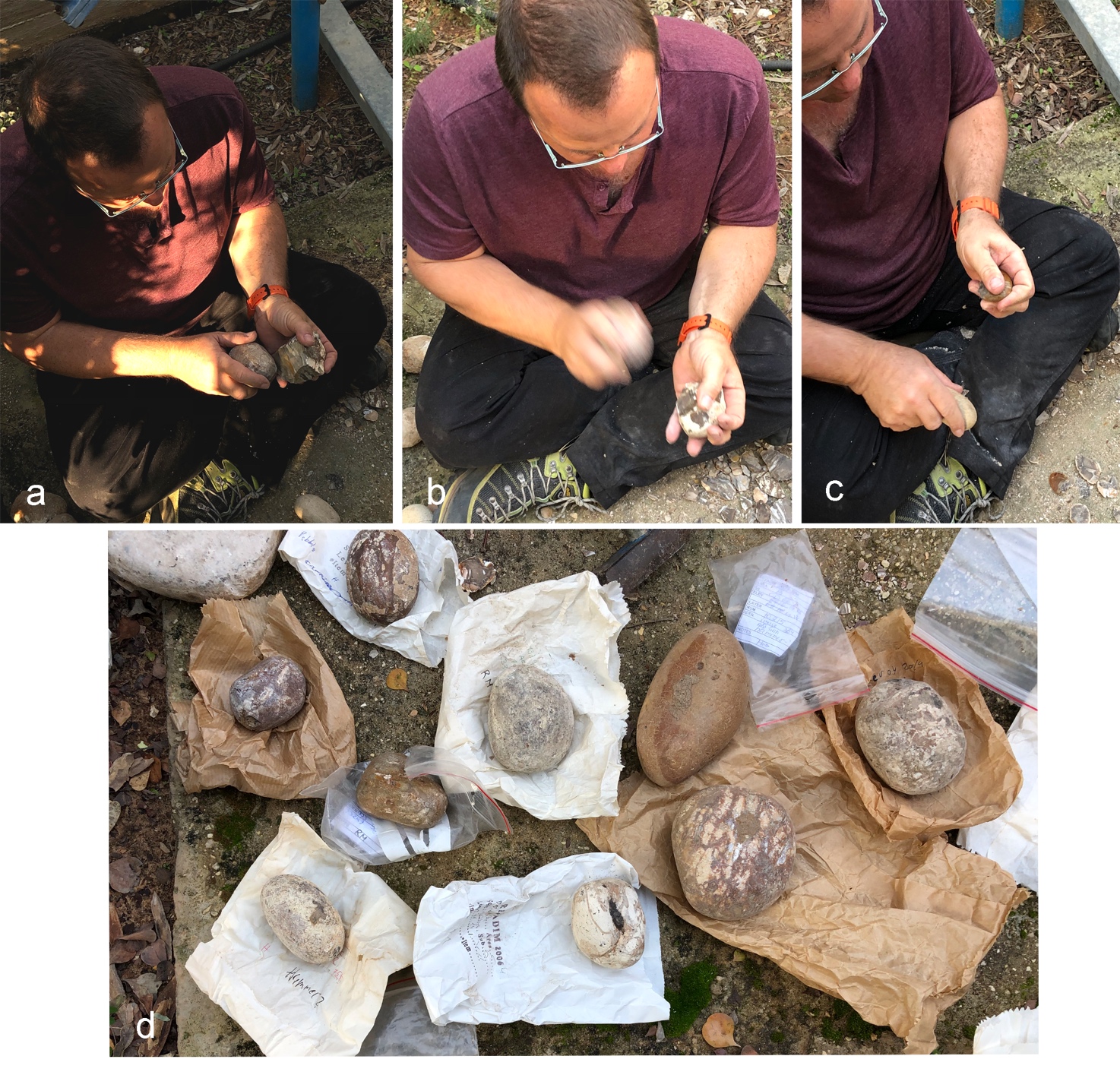
**Fig S4. Experimental manufacturing of chopping replica.** a-c) Different stages in the shaping of the chopping tools by direct percussion with a hard hammerstone; d) Unmodified pebbles used to produce chopping tool replicas.


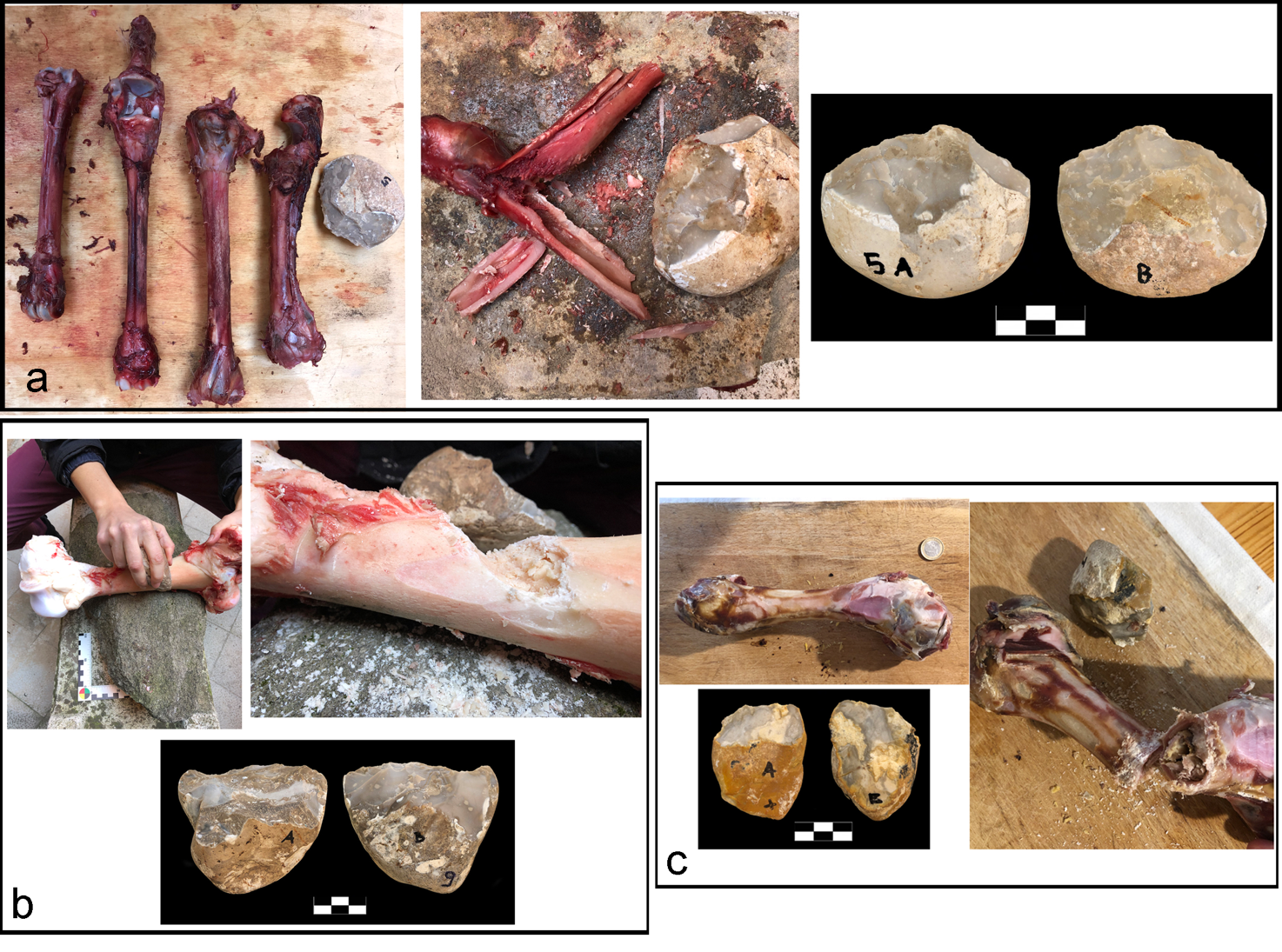


**Fig S5. Experimental bone breaking and different bone fractures observed in the process of marrow extraction.** a) Fresh roe deer bones (radio, ulna); b) Fresh cow bone (femur); c) Dehydrated pig bone with periosteum and meat (femur).


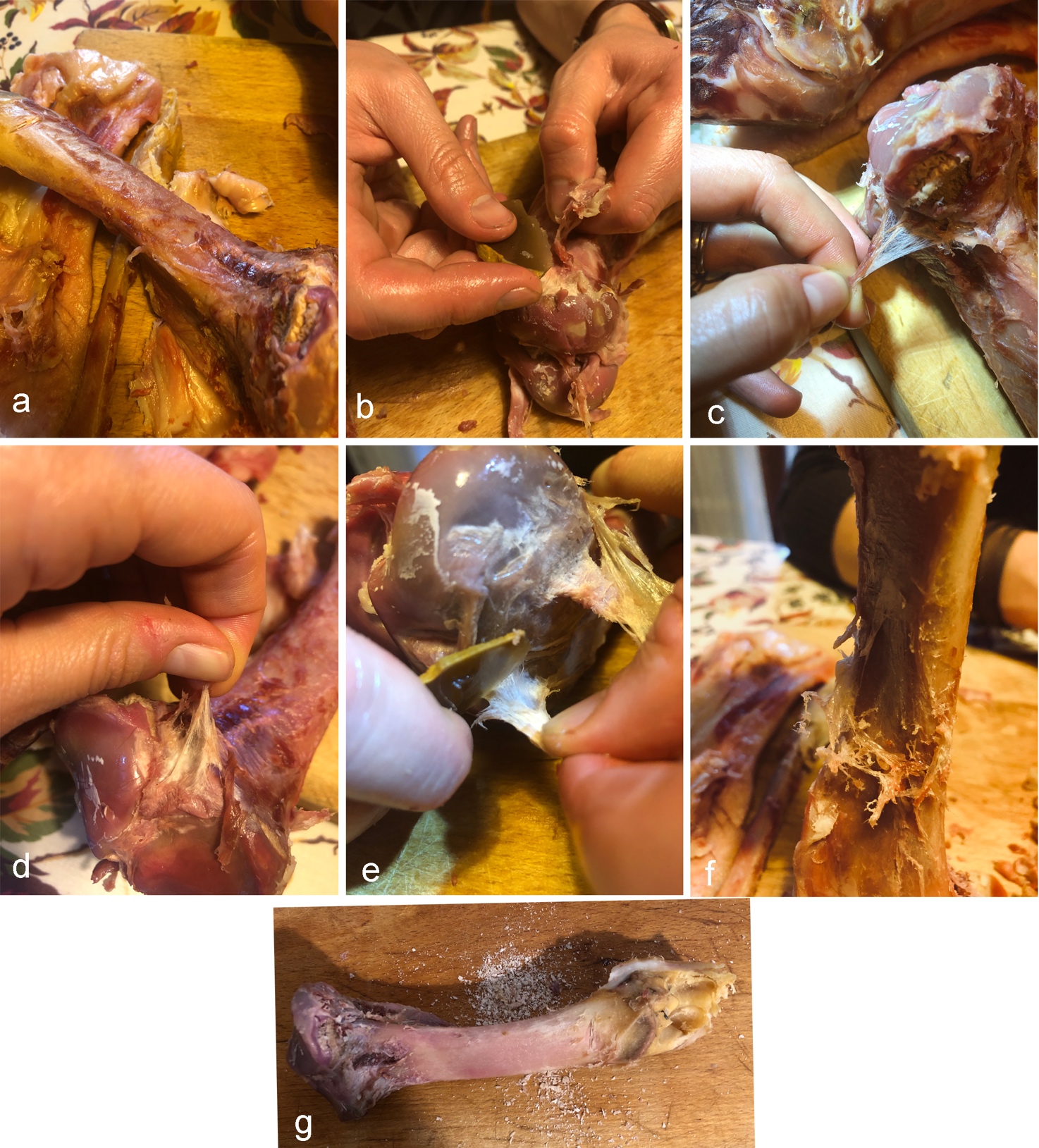


**Fig S6. Different stages in the process of cleaning meat and periosteum from a dehydrated pig bone.** a) Dehydrated bone still covered by fat, meat and periosteum; b-e) Cutting activities for removing dehydrated tissues from the bone with a cortical flake (note the frayed consistency of collagen fibers resulting from the loss of water in the tissues); i) Frayed collagen fibers partially attached to the bone; g) Cleaned bone ready for breaking.


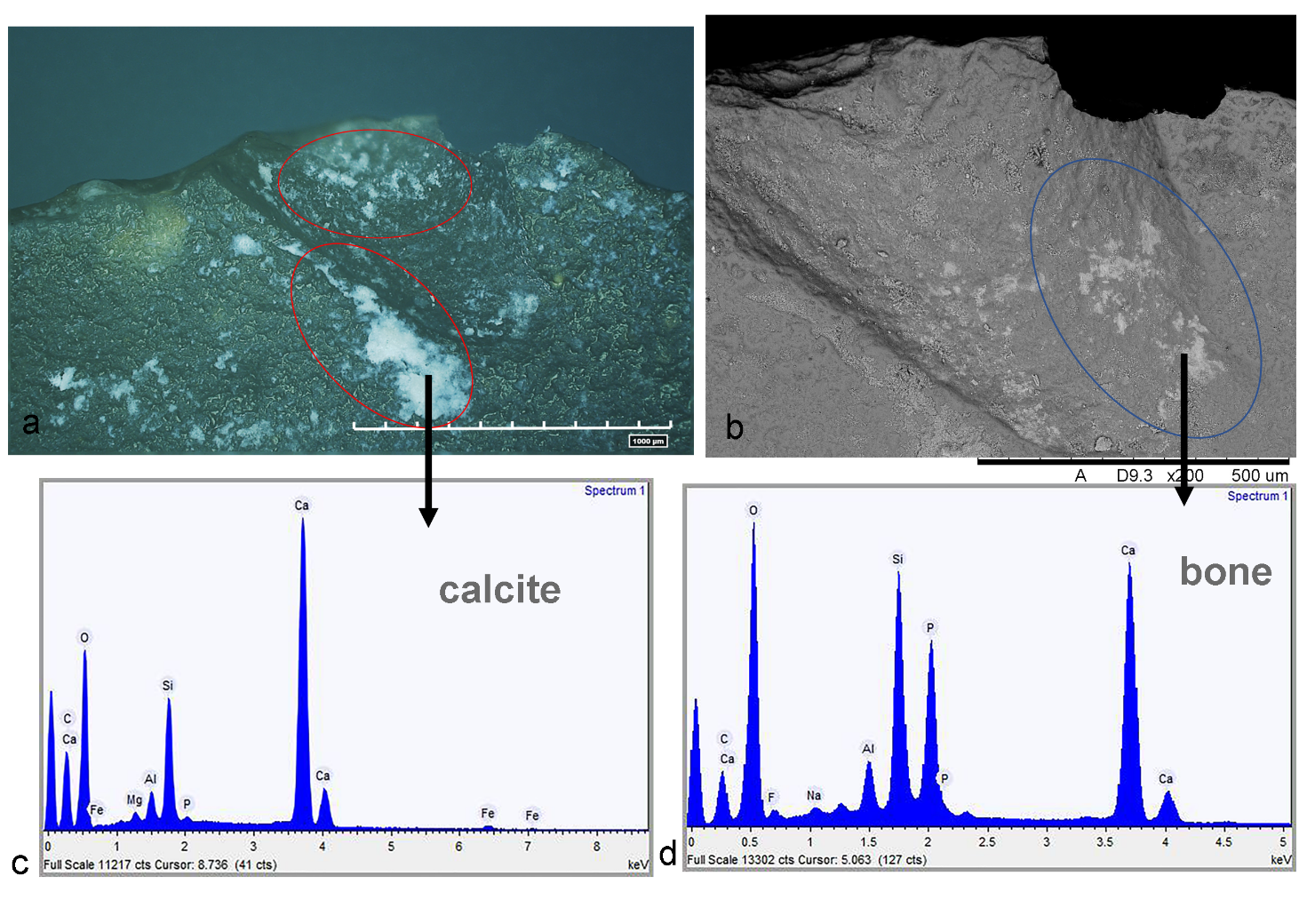


**Fig S7. Bone and calcite residues coexisting along the active edge of a tool in layer C3 at Revadim.** a) Scarring of the edge exhibiting whitish accumulations along the active edge; b) SEM micro-graph of the same edge portion showing topographical distinctive features for calcite and bone residue; c) Elemental analysis of calcite accumulation showing a notable major presence of calcium carbonate; d) Elemental analysis of bone showing the Ca/P at the proper ratio (~ 2:1).

**
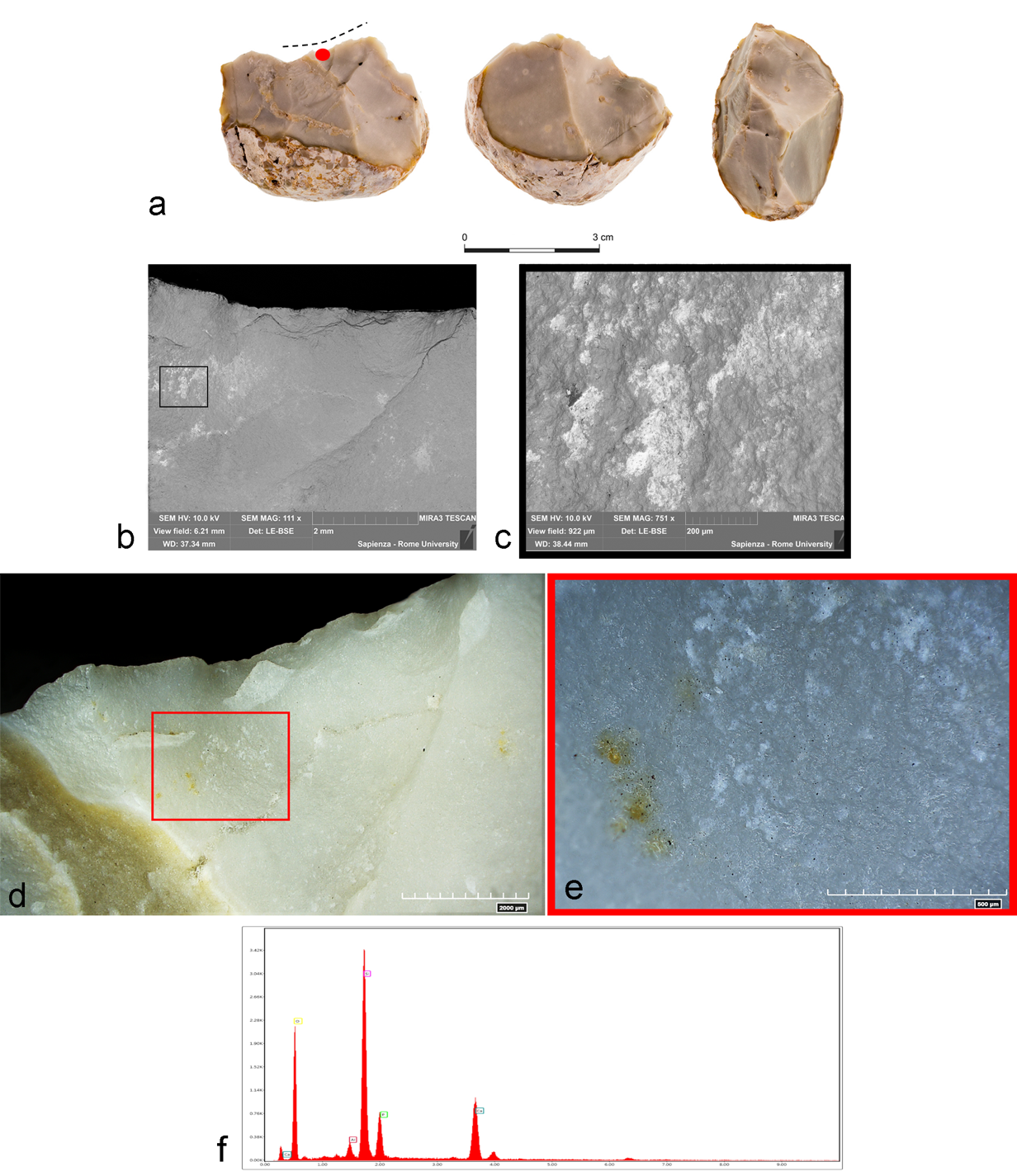
**

**Fig S8. Archaeological chopping tool with related use-wear and residue results.** a) Specimen #5 AV16a 71.03-71.01; b-c) SEM images showing edge damage associated with patches of bone residues along the functional edge; d-e) Spots of bone and fat matter spread just below the active edge and related close-up image (red square); f) SEM-EDX spectrum showing the bone diagnostic peak of calcium and phosphorus. Red dot indicates the area of residue, dotted line indicates the functional area.


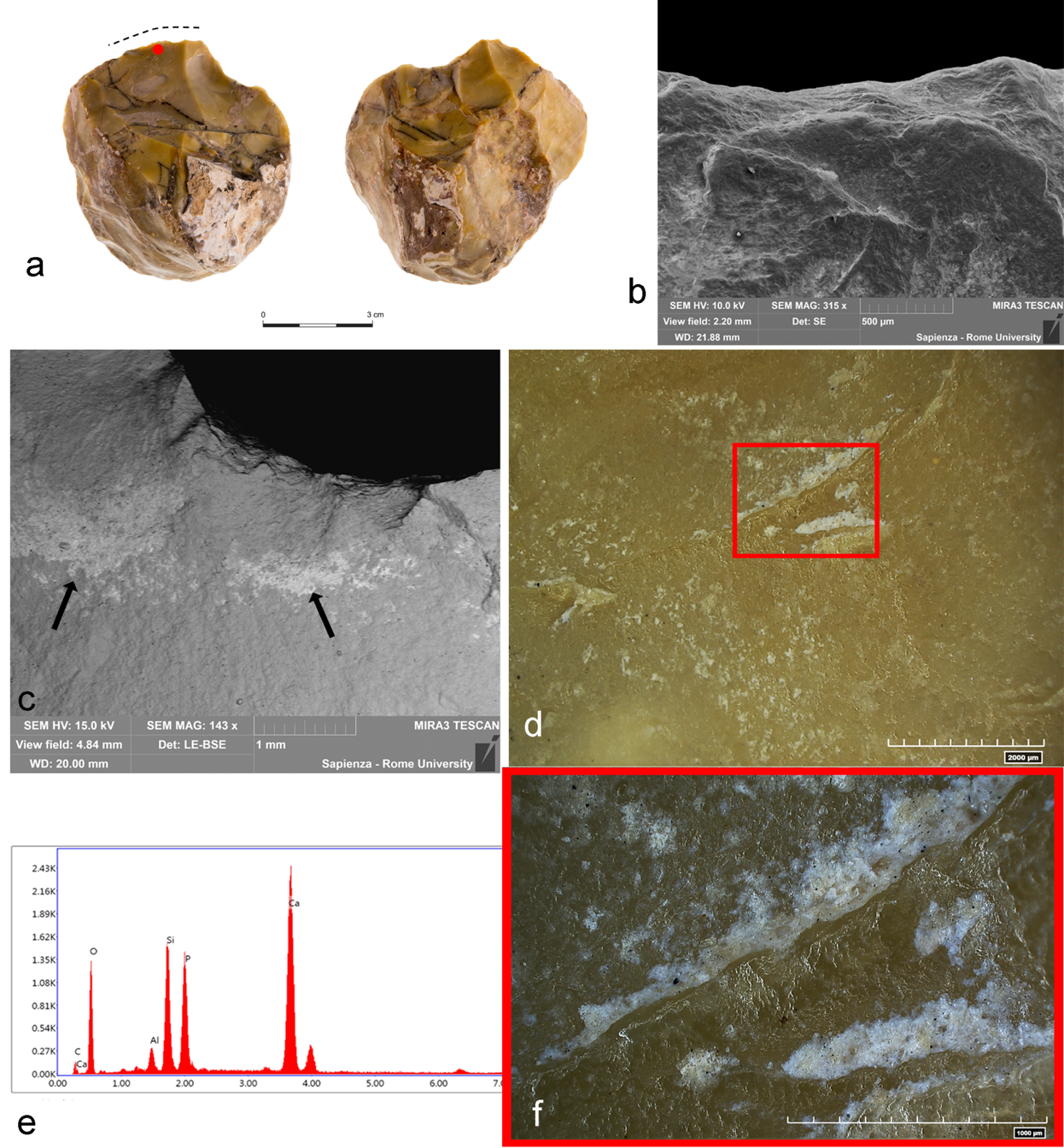


**Fig S9. Archaeological chopping tool with related use-wear and residue results.** a) Specimen #9AR16d 71.95-71.07; b, c) SEM image showing the edge rounding and scarring on the functional area and related bone residues (black arrows); d, f) Accumulation of bone mixed with fat matter inside technological scars located just below the edge and related close-up image (red square); e) SEM-EDX spectrum showing the bone diagnostic peak of calcium and phosphorus. Red dot indicates the area of residue, dotted line indicates the functional area.


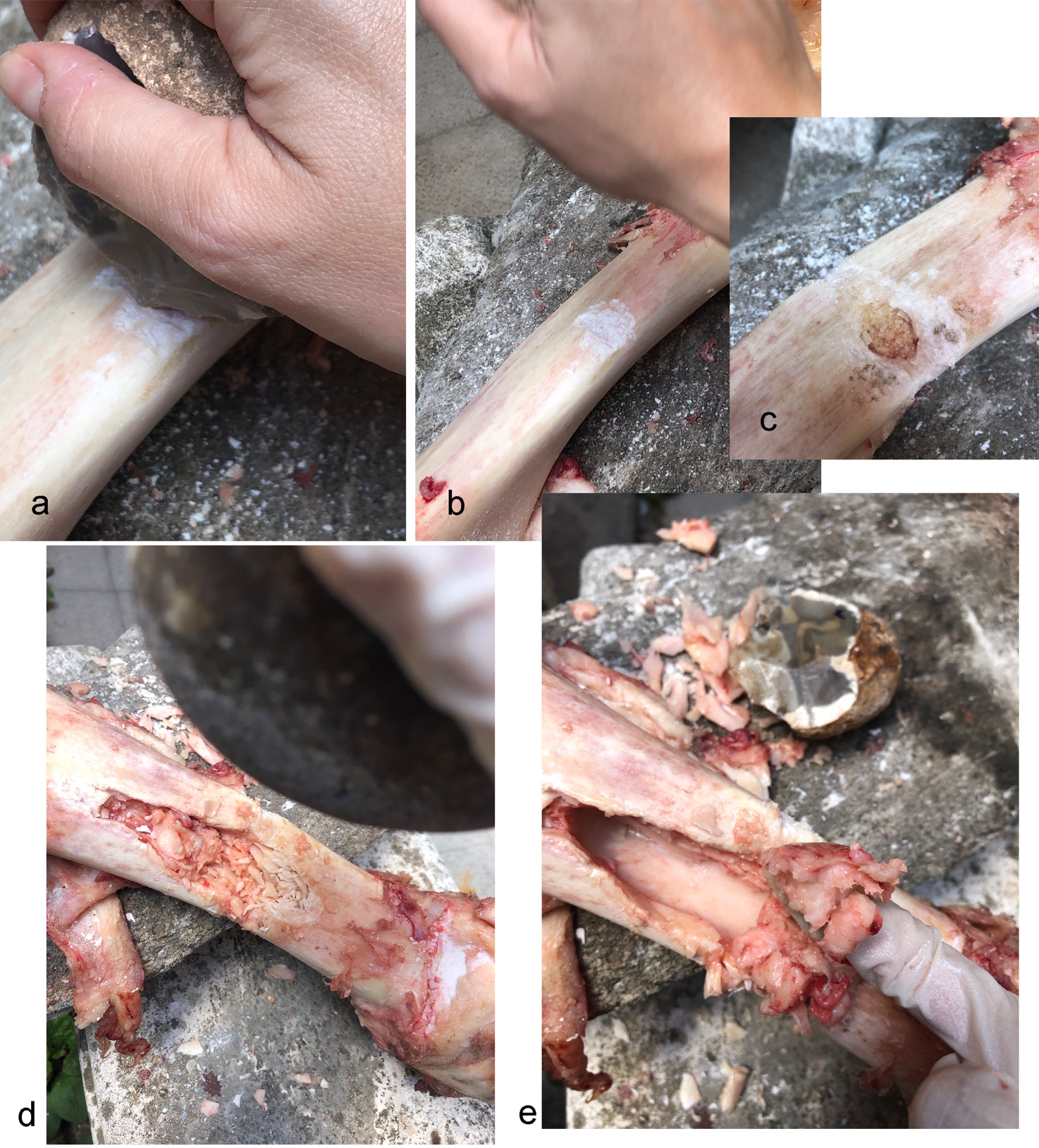


**Fig S10. Sequential experimental stages in the breaking of a cow tibia with a chopping tool assisted by a hard hammerstone.** a) Initial process of bone breaking with the chopping tool; b) Bone fracture starts to be delineated; c) The deep cut reaches the marrow; d) A hard hammerstone is used to longitudinally open the fracture; e) The marrow is collected through the long fracture.


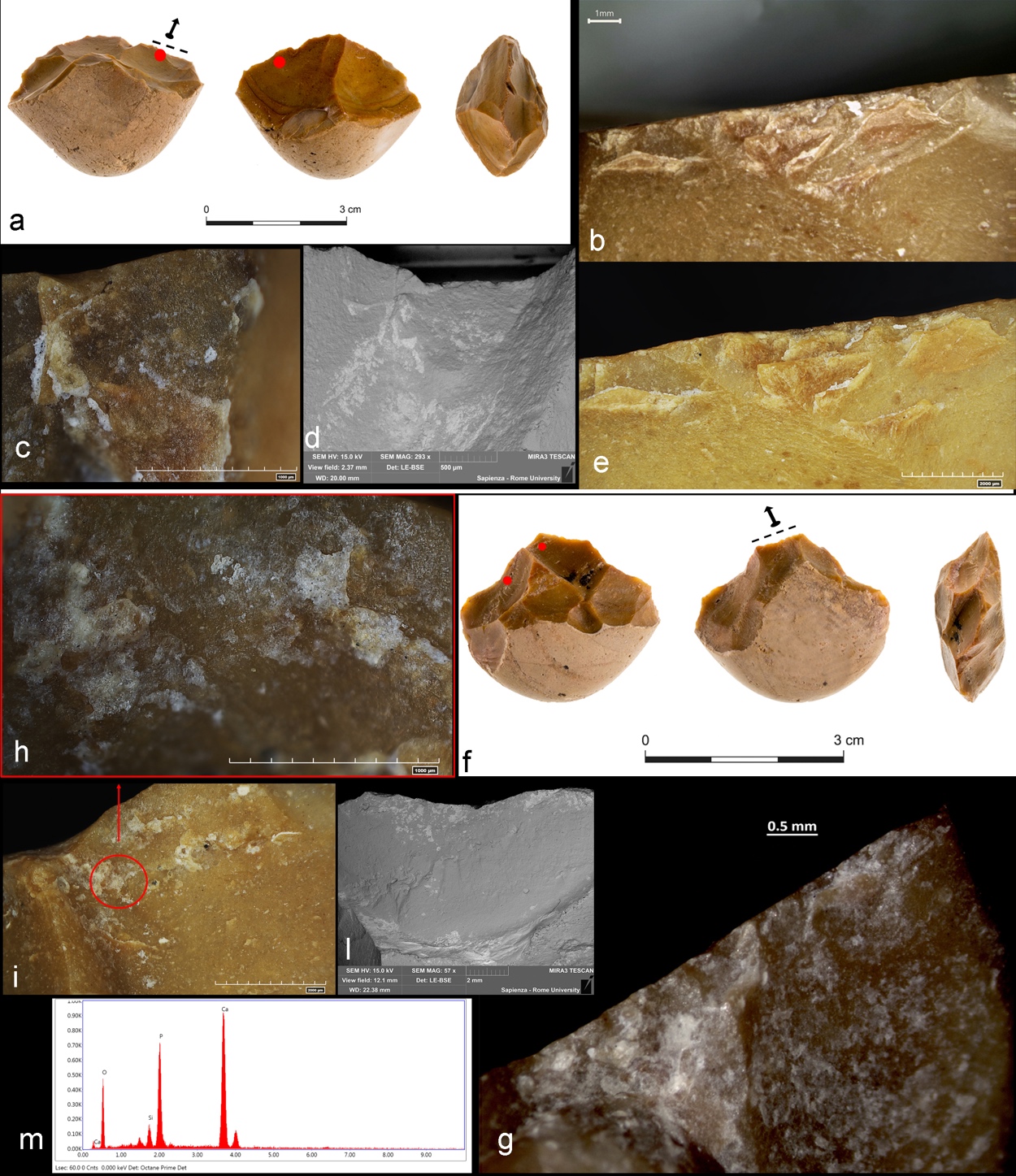


**Fig S11. Archaeological chopping tools with related use-wear and residue results.** a) Specimen #15 AS15a 71.15-71.07; b) Edge damage resulting from an activity in transversal motion; c-e) Patches of bone micro-residues smeared and entrapped in the edge scars along the functional edge; f) Specimen #32 AR14b71.13-71.11; g) Edge damage resulting from a transversal motion activity; h-i) Patches of bone micro-residues smeared along the functional edge and related close-up (red square); l) SEM image showing bone micro-residues; m) SEM-EDX spectrum showing the bone diagnostic peak of calcium and phosphorus. Red dots indicate the area of residue, dotted lines indicate the functional area.

Use-wear on experimental chopping tools

At the microscopic level, the experimental chopping tools exhibited visible damage on their edges after the chopping experiments (Fig 12 in S1 File). In two cases, the load and the force exerted with the tool on the bone caused part of the edge to fracture, resulting in the removal of small flint flakes and fragments.

When the edge outline is preserved, crushing of the pointed edge areas, compressions, and rounding of the edge are clearly visible (Fig 13A, D, F in S1 File). At high magnification, the compressions appear as overlapped deep scars with a stepped termination and rounded outlines **(**Fig 13B, C, E in S1 File). Their distribution along the edge is localized around the impact point, which is usually the central area of the outer edge.

Two types of polish are recognizable: smooth domed bone microwear polish (Fig 14A, B in S1 File) and smooth flat bone microwear with dense parallel striations perpendicularly oriented to the edge (Fig 14C in S1 File). The orientation of the striations and features of the wear traces are consistent with chopping bones. Polish distribution is not continuous along the edge but can be observed on the points of greatest impact

Micro-residue on experimental chopping tools

Bone-chopping activity resulted in significant splintering and shattering of the bone, leaving a large number of bone particles around the area of contact with the tool. Consequently, bone was smeared over the lithic surface, especially during the processing of the dehydrated bones (Fig 15A, B).

We observed two types of residue appearance and distribution on the chopping tool surfaces. In all the experiments during which bone was processed while still covered by fat, cartilage and periosteum, the quantity of bone residue along the edge of the tool was small and consisted mainly of collagen tissues and fibers, fat, and bloody matter. Bone is mostly visible as an isolated or grouped dense mass of tissues, amorphous in shape and white-yellow in color, mixed with light brownish collagen fibers and a mixture of fresh residues (fat, periosteum membrane, blood) clustered in bands or patches along the edge (Fig 15C-E). Residues were localized on top of the outer edge, inside the damaged edge, but also far from the edge and at the extremity of the chopping tool, where the contact with the worked material was only accidental.

When bone was instead processed after the periosteum was removed, we observed different residue patterns. In these cases, no collagen fibers and tissues were identified, while the utilized edge of the tool and a wide area below it were covered by a dense, spread mass of bony powder along with clustered masses of bony tissues appearing translucent and whitish in color (Fig 15F-I).

Finally, our experiments also included a test to evaluate the morphological modification of animal residues after undergoing diagenesis. Although this would usually require a different set of controlled experiments, preferably conducted in conditions similar to the archaeological context, our goal here was not to replicate the taphonomic processes which affected the residues in the sediment of Revadim. We rather wanted to test how fresh residues such as marrow and bony tissues react and change their physical properties as a result of a microbial attack following a period of interment. Chopping tool replica CT#9 was chosen for the burial experiment because of the massive presence of fresh marrow and bone residues attached to the edge after the bone-chopping activity (Fig 16A, C). The artifact was buried at a depth of 20 cm in a universal potting soil mixed with sand in an open-air location for two months, during which the soil was subjected to both natural precipitation and anthropic irrigation.

Once retrieved from the soil, the item was lightly dry-brushed to remove soil particles and then analyzed under a stereo and digital microscope. With the naked eye and at low magnifications, we observed a high quantity of bony tissues and organic compounds spread over a large band along the chopping edge (Fig 16B). The white fresh marrow fat deposit was turned into a dry and semi-hard yellow crust with green shadows and several worms tunneling inside, indicating that the soil was biologically active and that a microbial attack had begun (Fig 16C, D). We thus performed a deep wash in deionized water to remove the larger accumulations as well as the worms. Later, using OLM, we recorded yellow-brown opaque (translucent under high magnification and polarized light) residues resulting from the biodegradation of marrow adipose tissues clustered with whitish and white-yellow residues of bony tissues organized both as masses filling the edge-scar depressions and as a dense accumulation of small particles (suggesting a powdery appearance) covering a large portion of the edge (Fig 16E, F).


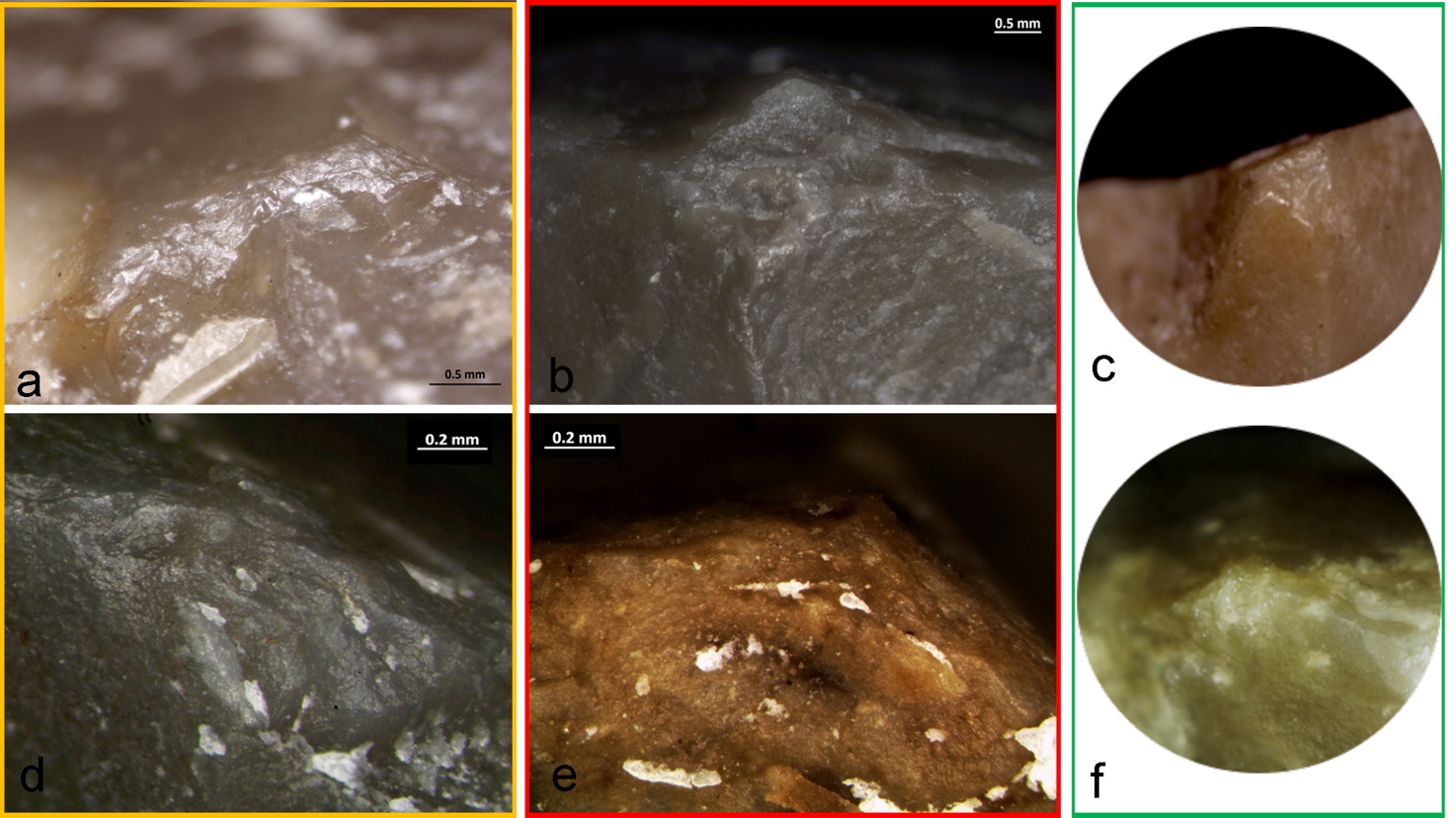


**Fig S12. Comparison between archaeological and experimental edge damage.** a-c) Archaeological edge damage; d-f) Experimental edge damage.


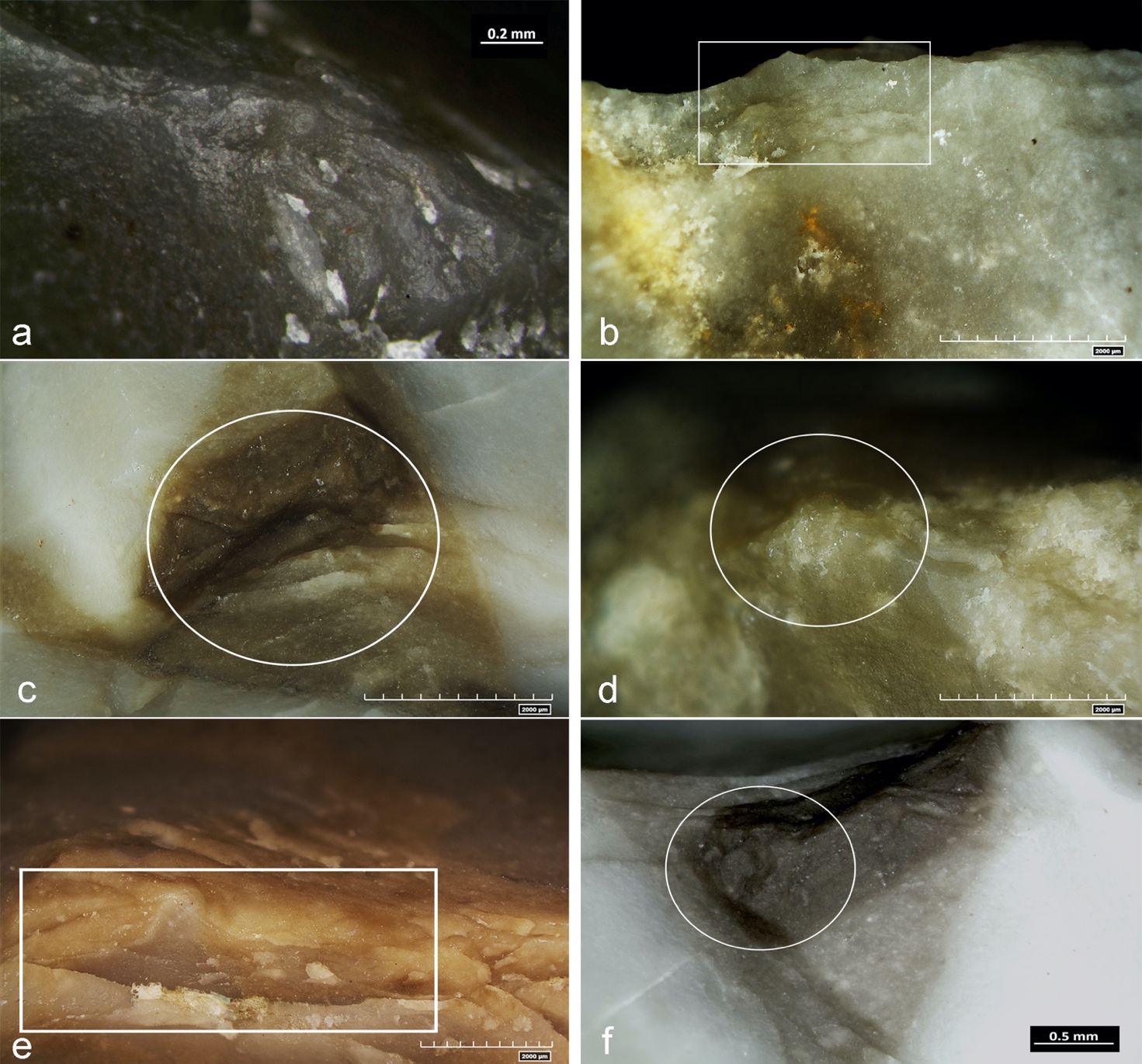


**Fig S13. Experimental edge damage observed after bone chopping activity.** a) Compression, scarring and rounding of the edge; b-c) Stepped overlapping scars; d) Crushing of the pointed asperities; e) Deep scar with stepped termination; f) Crushing of the pointed asperities.


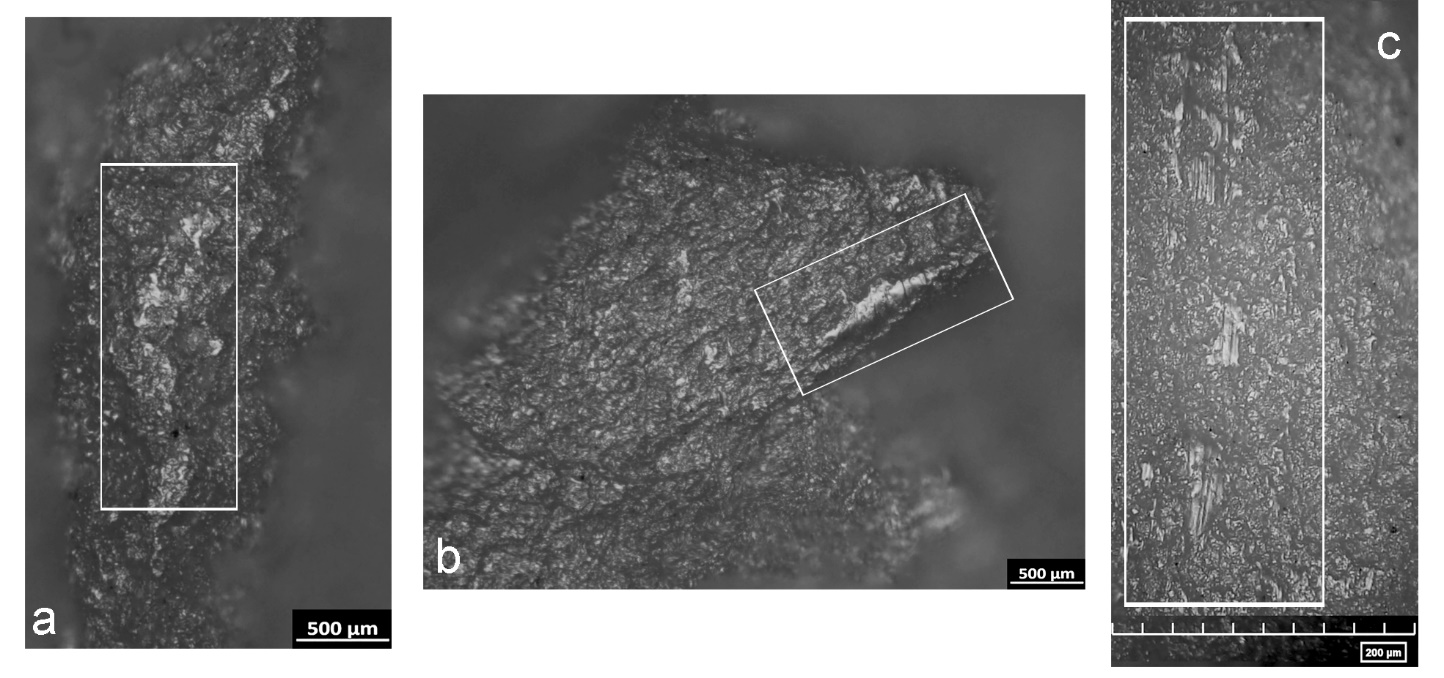


**Fig S14. Experimental micro-wear observed after the bone chopping activity.** a-c) Bone polish.


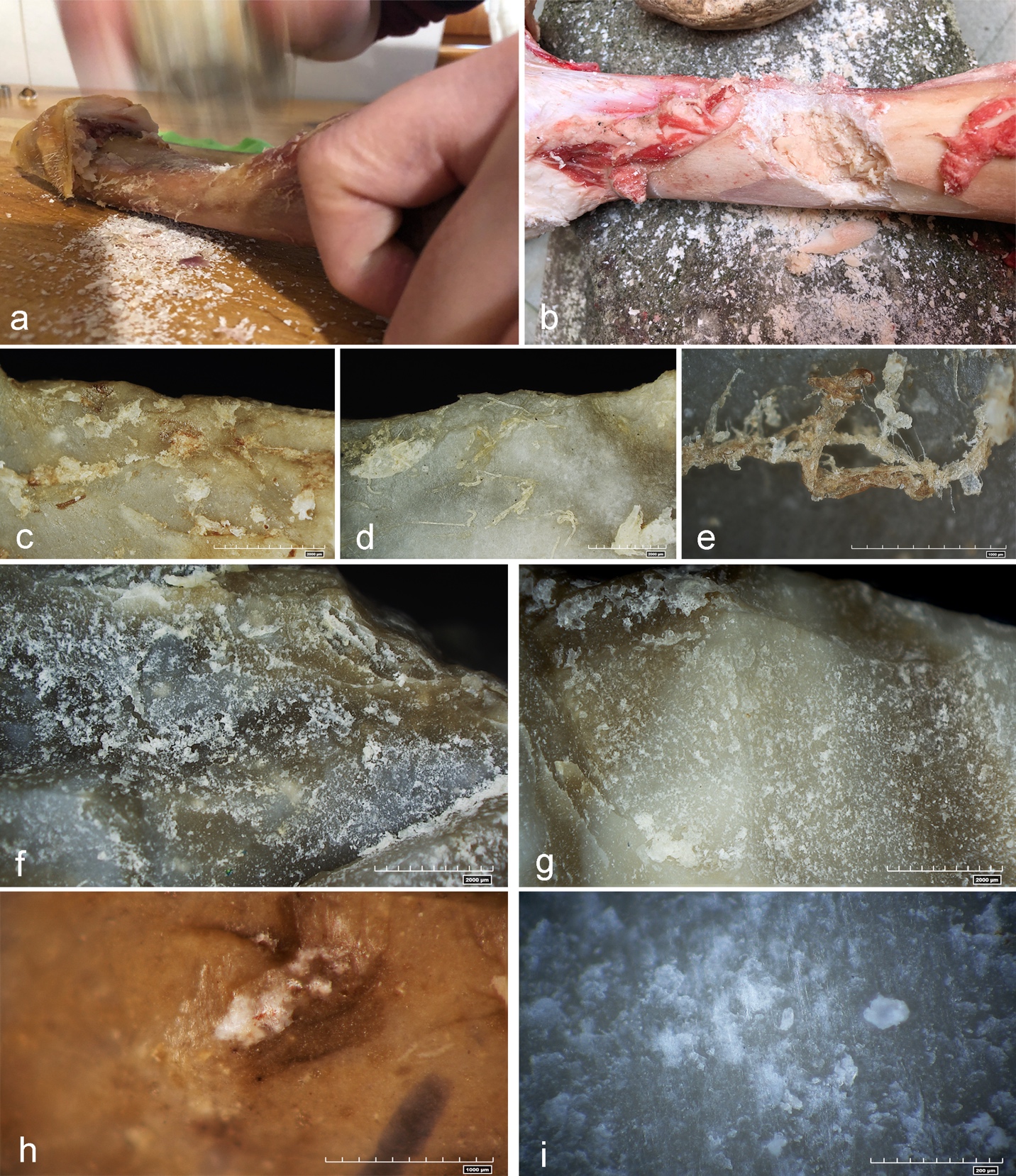


**Fig S15. Residues observed after experimental bone breaking activity.** a) Breaking of dehydrated bone (note the large number of bone splinters around the bone; b) Breaking of fresh bone (note the large number of bone splinters around the bone); c-e) Bone and collagen fibers adhering to tool surfaces after breaking bones still covered by periosteum, meat and collagen tissues (note the distribution of bone clumps and fiber residues massed along the edge); f-g) Bone residues adhering to the tool surfaces after breaking bones without periosteum (note the distribution of the bone micro-residues which appear as a veil of bony powder covering a large area of the tool surface; h-i) Close-up view of the bone residues showing a glossy and striated appearance.


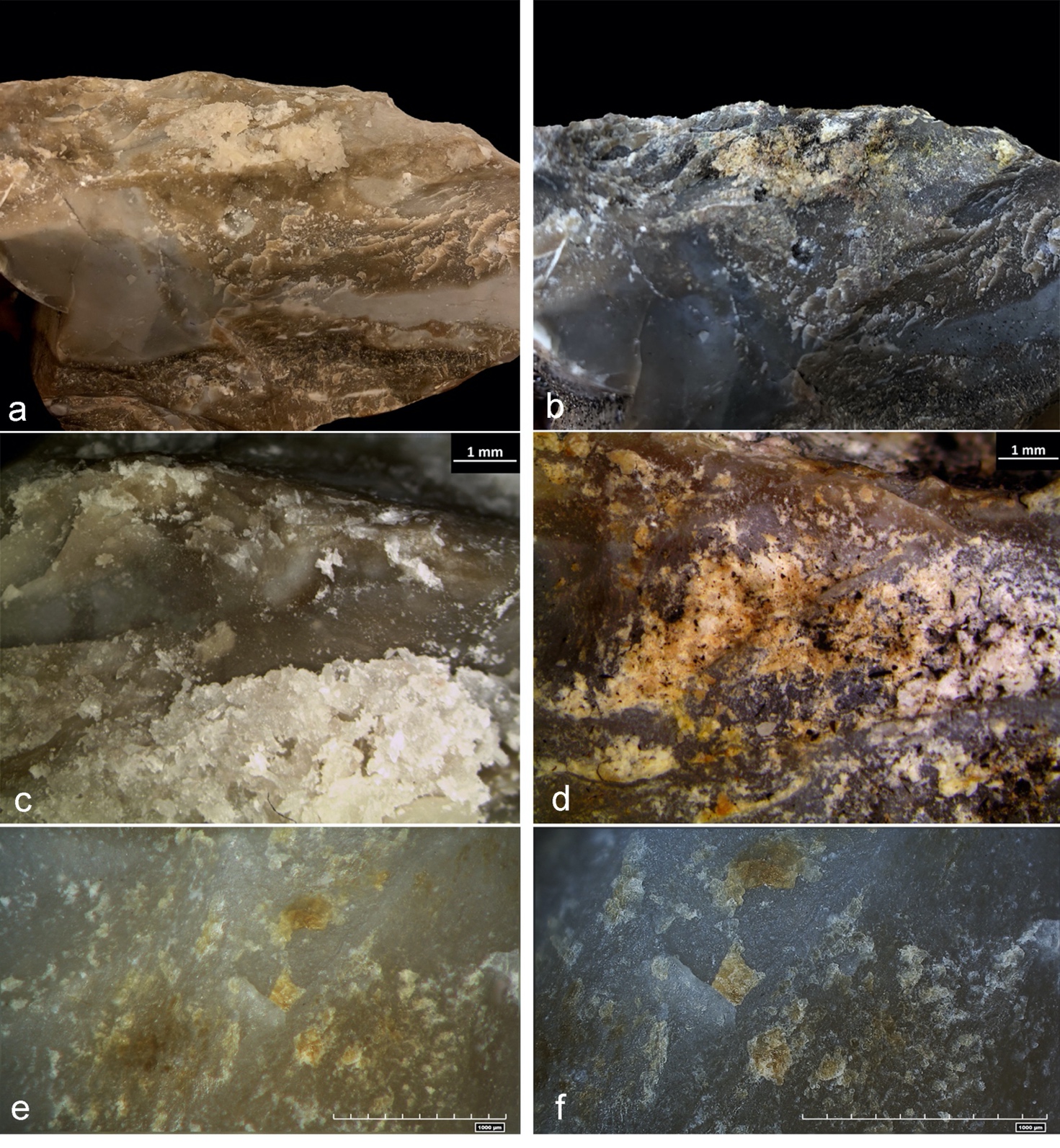


**Fig S16. Bone and marrow residues after undergoing diagenesis.** a) Experimental chopping tool used to process a fresh cow femur (note the accumulation of marrow in the middle of the utilized edge); b) Experimental chopping tool after interment (note the change in appearance of the residue); c-d) Close-up view of marrow residue before and after the interment; e-f) Close-up of the marrow residues after washing procedure (note the change in coloration and appearance from whitish to yellowish shades).

| **Group of chopping tools** | **Quantity** | **Average number of scars** | **Average weight (in grams)** | **Average length (in cm)** | **Average thickness (in cm)** | **Average width (in cm)** |
| --- | --- | --- | --- | --- | --- | --- |
| 1 | 25 | 6.7 | 47.4 | 4.1 | 1.5 | 4.5 |
| 2 | 16 | 6.8 | 107.6 | 4.9 | 3.3 | 5.6 |
| 3 | 9 | 7.4 | 222.0 | 5.9 | 4.2 | 6.6 |
| 4 | 3 | 8.7 | 125.3 | 4.5 | 4.1 | 5.4 |
| **Total** | **53** | **7.0** | **102.6** | **4.7** | **2.7** | **5.3** |

**Table S1:** The chopping tools, with their average attributes.
